# Extracellular vesicles: A new therapeutic drug for nerve injury repair

**DOI:** 10.4103/NRR.NRR-D-25-00133

**Published:** 2025-12-30

**Authors:** Jinyi Cai, Chongkang Ren, Changshui Wang, Songmin Shen, Biao Xu, Changmeng Cui

**Affiliations:** 1 Jining Medical University, Jining, Shandong Province, China; 2 Department of Neurosurgery, The First Bethune Hospital of Jilin University, Changchun, Jilin Province, China; 3 Department of Neurosurgery, Affiliated Hospital of Jining Medical University, Jining Medical University, Jining, Shandong Province, China

**Keywords:** brain diseases, central nervous system diseases, exosomes, extracellular vesicles, mesenchymal stem cells, microglia, nerve regeneration, neurodegenerative diseases, spinal cord injuries

## Abstract

Extracellular vesicles are membranous structures actively released by cells. They efficiently mediate intercellular communication and play a role in reshaping the microenvironment. Their diverse bioactive cargo, including miRNAs, proteins, and lipids, along with their ability to cross the blood–brain barrier, holds broad therapeutic promise in the field of neuroregeneration. Current research focuses on exploring the mechanisms of action and clinical translational potential of extracellular vesicles from different sources, including exosomes derived from mesenchymal stem cells, neural stem cells, neurons, and vascular cells, in neural repair. Key issues restricting clinical translation include the lack of standardized isolation and characterization methods, insufficient dose determination and bioavailability assessment, and inadequate evidence for long-term safety. This review systematically summarizes the therapeutic research progress of extracellular vesicles from various sources in neurological disorders such as brain injury, spinal cord injury, and neurodegenerative diseases. It provides a comprehensive overview of the key molecular regulatory networks involving extracellular vesicles, including the regulation of neuroinflammation, axonal regeneration, mitochondrial function and metabolic homeostasis, as well as structural and functional support of the neurovascular unit. In addition, this review assesses the feasibility of extracellular vesicles as drug delivery vehicles. In summary, extracellular vesicles constitute a dynamic and multi-pathway regulatory network. A thorough elucidation of their mechanisms of action is expected to facilitate the development of novel therapeutic strategies in the field of neural repair and provide new perspectives and solutions for precision medicine.

**Facts**

• Exosomes from multiple sources drive neural repair through four axes: modulation of neuroinflammation, axonal regeneration, maintenance of mitochondrial/metabolic homeostasis, and support of the neurovascular unit.

• In brain injury, spinal cord injury, and neurodegenerative diseases, extracellular vesicles demonstrate potential as delivery vehicles capable of crossing the blood–brain barrier, although their delivery efficiency still needs further optimization.

• The miRNAs and proteins carried by extracellular vesicles exert functional effects related to neural regeneration by targeting specific molecular pathways and targets.

**Open questions**

• How can standardized methods for the isolation and characterization of extracellular vesicles suitable for the central nervous system be established, along with comparable dosing quantification standards?

• Which source characteristics or surface markers are most predictive of the *in vivo* targeting and functional effects of extracellular vesicles in the human central nervous system?

• How can the therapeutic effects and durability of benefits of extracellular vesicles be validated under conditions of long-term administration?

## Introduction

Extracellular vesicles (EVs) possess a lipid bilayer envelope that enables the transport of a variety of bioactive molecules, including miRNAs and proteins, between cells (Jeppesen et al., 2023; Nowak et al., 2023; Kumar et al., 2024). Exosomes from different sources are the most intensively studied EV subtype, with unique biogenesis mechanisms that enable them to effectively mediate intercellular signaling and regulate the microenvironment. Consequently, they have become a core focus of research on neural regeneration and repair.

**Figure 1:**
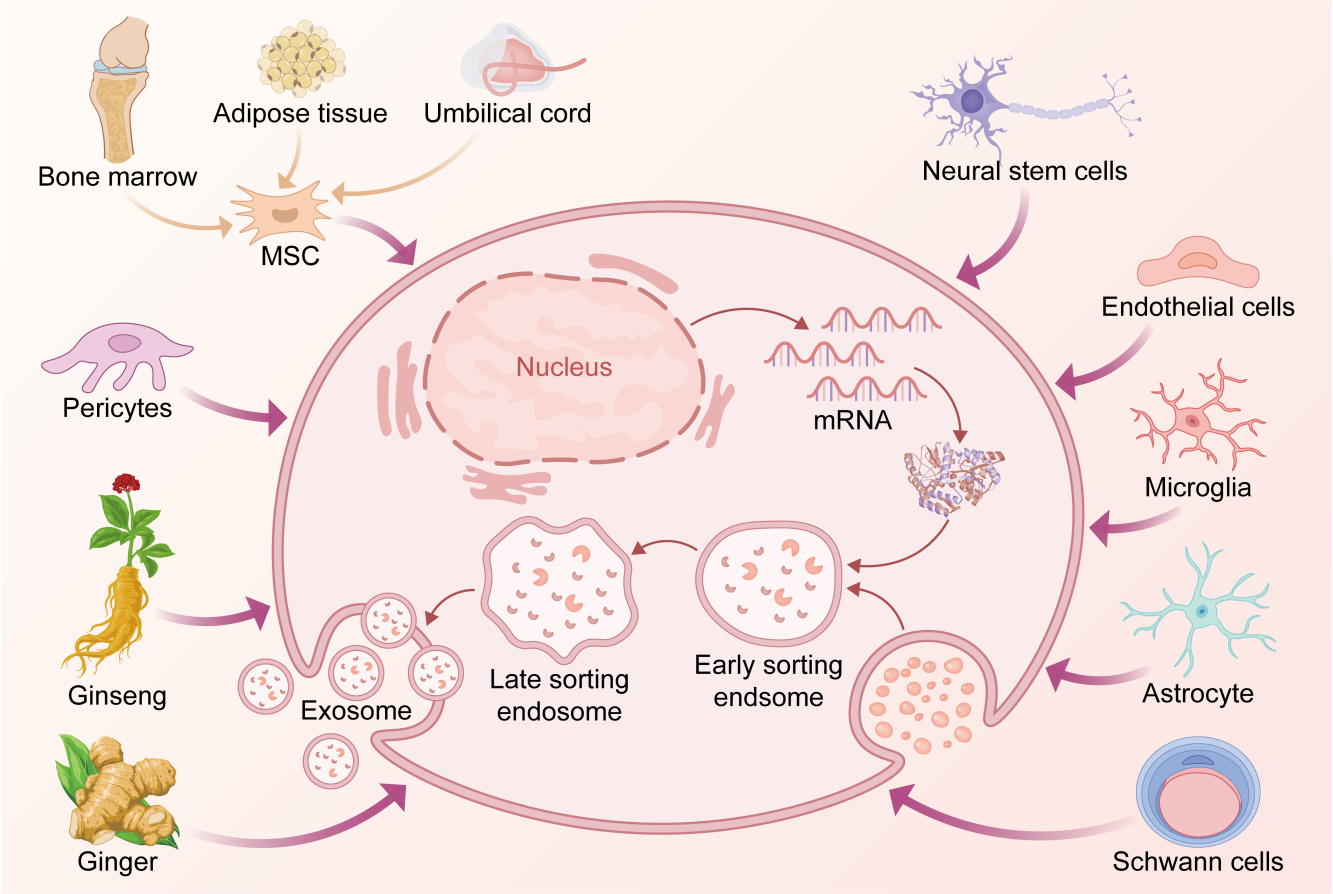
Cells from various origins, including plant cells, vascular endothelial cells, neural cells, and mesenchymal stem cells, can secrete extracellular vesicles. These EVs are released into the body via exocytosis and carry a range of bioactive molecules that facilitate intercellular communication and mediate functional effects. MSC: Mesenchymal stem cells.

In recent years, with the advancement of neuroregenerative science, small extracellular vesicles (SEVs) have emerged as a novel cell-free therapeutic strategy for addressing central nervous system (CNS) diseases, owing to their low immunogenicity, excellent biostability, and ability to cross the blood–brain barrier (BBB) (Tenchov et al., 2022). For example, stem cell-derived exosomes can deliver miRNAs and neurotrophic factors, which regulate neural stem cell proliferation, promote axonal regeneration, and ameliorate the neuroinflammatory microenvironment (N V Lakshmi Kavya et al., 2022; Lin et al., 2022; Huerta et al., 2023; Ghosh and Pearse, 2024). However, existing studies have shown that exosomes from different sources exhibit significant differences in cargo composition, signaling pathway activation patterns, and downstream effects, with some even exhibiting opposing effects under certain pathological conditions. Furthermore, the lack of a unified theoretical framework for their mechanisms of action poses numerous challenges to clinical translation. By deeply analyzing the interactions between EVs and neurological diseases, researchers are exploring new perspectives on neuroregeneration and repair and identifying promising intervention targets (**Figure 1**).

## Literature Retrieval Strategy

Studies cited in this review were retrieved from PubMed and Web of Science. The majority of the selected studies (77% of all references) were published between January 2021 and August 2025. A combination of MeSH and free-text search terms was performed, using terms related to “Extracellular vesicles,” “Exosomes,” “Mesenchymal stem cells,” “Endothelial cells,” “Neural cells,” “Spinal cord diseases,” “Brain diseases,” “Neurodegenerative diseases,” “Neural regeneration,” and “microvesicles,” combined with Boolean operators. Inclusion criteria included: (i) peer-reviewed original research or review articles; (ii) a focus on the effects of exosomes from different sources on neural regeneration; and (iii) English-language publications. Exclusion criteria included conference abstracts, editorials, case reports, letters, non-English publications, and retracted articles.

## Extracellular Vesicles

**Figure 2:**
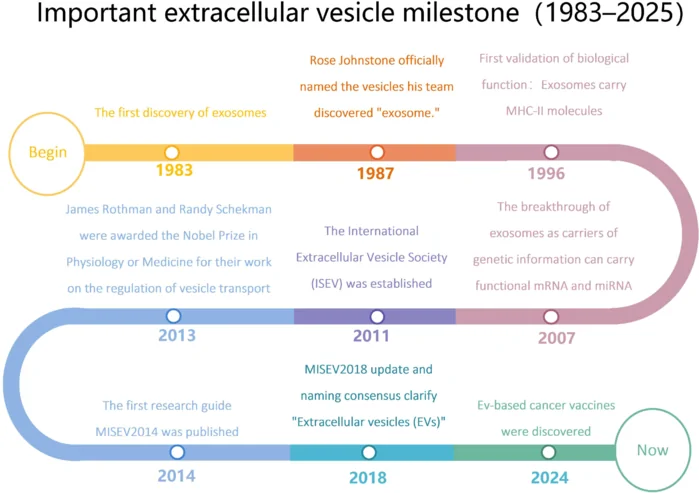
Milestones in EV research. This figure highlights key milestones in the field of extracellular vesicle research, including the discovery of exosomes in 1983, the establishment of the International Society for Extracellular Vesicles (ISEV) in 2011, which considerably accelerated progress in the field, the publication of the first research guidelines in 2014, and the nomenclature update in 2018 that further standardized and advanced research practices. Additionally, recent years have seen continuous breakthroughs in this area (Couch et al., 2021; Zhang et al., 2024c).

EVs are membrane-bound structures released by cells into the extracellular environment, playing a key role in intercellular communication (**Figure 2**; Witwer and Théry, 2019; Chang et al., 2021; Hade et al., 2021; Zhang et al., 2024b). By transporting bioactive molecules such as proteins, lipids, and RNAs, EVs regulate signal transduction in recipient cells, influencing gene expression, metabolic states, and cellular functions, thereby contributing to various physiological and pathological processes (Yáñez-Mó et al., 2015; Yang et al., 2024a). In recent years, significant progress has been made in EV research across various fields, including neuroscience, immune regulation, and disease diagnosis. In particular, EVs have garnered increasing attention as critical mediators of intercellular signaling in the development and repair of neurological disorders (Kim et al., 2022). EVs are primarily categorized into three types based on their biological origin: exosomes, microvesicles, and apoptotic bodies, each with distinct biogenesis pathways and functional properties (Davidson et al., 2023). They are also typically classified by size, with small EVs being less than 200 nm and large EVs greater than 200 nm (**Additional Table 1**; Welsh et al., 2024).

**Additional Table 1:** EV nomenclature

Term	Definition
EVs	Particles released by cells, enclosed by a lipid bilayer, and incapable of autonomous replication (i.e., do not contain a functional nucleus).
Small EVs	Based on measured particle diameter after isolation, small EVs are typically < 200 nm in diameter.
Large EVs	Based on measured particle diameter after isolation, large EVs are typically > 200 nm in diameter.
Exosome	Vesicles originating from the endosomal system, explicitly denoting subcellular origin. Exosomes constitute a subtype of small EVs. The intraluminal vesicles of endosomes typically have diameters < 200 nm.
Ectosome	Vesicles originating from the plasma membrane, explicitly indicating subcellular origin. Their size range can be broad, encompassing sizes comparable to those of exosomes.
Microvesicle	Vesicles originating from the plasma membrane have historically been referred to as "large EVs" or even broadly as "all EVs".

EVs: Extracellular vesicles.

Exosomes are considered as SEVs and possess unique properties regarding their biogenic pathways, molecular composition, and functional characteristics, which distinguish them from microvesicles and apoptotic bodies (van Niel et al., 2018; Kim et al., 2024). The biogenesis of exosomes relies on the endocytic-multivesicular body pathway, rather than directly budding from the cell membrane or being released during apoptosis (Pegtel and Gould, 2019). During this process, primary endosomes containing numerous endocytic vesicles fuse with lysosomes for degradation, while others fuse with the cell membrane to release exosomes. The distinct biogenic pathways of exosomes shape their unique molecular composition, which includes TSG101, ALIX, and tetraspanins such as CD63, CD81, and CD9, widely recognized as characteristic biological markers of exosomes (Kowal et al., 2016). In contrast, microvesicles are generated through asymmetric changes in the cell membrane regulated by the actin-myosin system, while apoptotic bodies typically contain intact organelles and DNA fragments from apoptotic cells. Exosomes exhibit significant differences in molecular composition and function compared with other EVs, which contributes to their classification and impacts their mechanisms of action in biological functions and disease regulation.

Although the different types of EVs exhibit obvious differences in biogenesis and molecular composition, all are rich in bioactive molecules, including proteins (such as heat shock proteins and integrins), lipids (such as phosphatidylserine and cholesterol), and nucleic acids (such as mRNAs, miRNAs, and lncRNAs). These components endow EVs with multiple functions in cellular signal transduction, inflammation control, tissue repair, and disease progression. SEVs in the CNS can regulate neuronal plasticity by transmitting miRNAs and proteins, while microvesicles play a role in vascular homeostasis and the inflammatory response (D’Acunzo et al., 2022). During immune regulation, apoptotic bodies are cleared by phagocytes and may impact the onset of autoimmune diseases. These findings indicate that EVs are not only key carriers of intercellular signal transduction but also hold potential application value in the occurrence, development, and treatment of various neurological diseases.

## Pathogenesis of Central Nervous System Disorder

The CNS, composed of the brain, spinal cord, and highly specialized neural networks, maintains homeostasis through multi-level signal transduction mechanisms (Schnatz et al., 2021; Mohanta et al., 2023). With the aging population, the incidence of CNS diseases continues to rise (Tu and Wang, 2023). The pathogenesis of these diseases typically involves the interaction of primary injury and secondary pathological responses, with significant pathological differences among various disease types.

Traumatic brain injury (TBI) is a prime example of a brain trauma-related disease, often caused by direct external forces acting on the brain (Blot et al., 2025). The pathogenesis includes primary injury, which involves mechanical damage to neurons, glial cells, and cerebral blood vessels caused by external forces, and secondary injury, which encompasses neuroinflammation (including microglial activation and the release of pro-inflammatory cytokines), disruption of the BBB, excitotoxicity (excessive glutamate leading to Ca^2+^ influx and mitochondrial damage), oxidative stress, caspase-dependent apoptosis, and necrosis. A notable example of long-term neurological changes is chronic traumatic encephalopathy, characterized by abnormal tau protein deposition and persistent neuronal network degeneration (VanItallie, 2019). Post-concussive syndrome is a common long-term symptom following TBI, characterized by persistent neuronal impairment (O’Day, 2024).

The pathogenesis of other CNS diseases can be briefly summarized as follows: Neurodegenerative diseases, such as Alzheimer’s disease (AD), are characterized by amyloid-β deposition and tau hyperphosphorylation, leading to significant impairment in information integration within the hippocampus and cortical neural networks (Zhang et al., 2021a; Gilbert et al., 2024). The core mechanism of Parkinson’s disease (PD) is the progressive loss of dopaminergic neurons in the substantia nigra of the midbrain, accompanied by abnormal aggregation of α-synuclein (Hall et al., 2014). Cerebrovascular diseases are characterized by ischemia-reperfusion (I/R) injury, blood-brain barrier disruption, and inflammatory cascades. Neoplastic diseases, such as gliomas, involve immune evasion and hypoxia-driven remodeling of the tumor microenvironment (Ali et al., 2025).

Overall, the pathogenesis of CNS diseases is characterized by multiple, interactive processes. Secondary injury following brain trauma is a key intervention target that requires urgent attention. In recent years, the continued advancement of neuroscience has led to exosome research providing new potential strategies for the treatment of CNS diseases (Liu et al., 2025; Spinelli et al., 2025). Exosome research not only offers novel biomarkers for the early diagnosis of CNS diseases but also opens new avenues for the development of future clinical treatment options (Holm et al., 2018).

## Extracellular Vesicles and Neurological Disorders: Interrelationships and Neuroprotective Mechanisms

### Stem cell–derived exosomes

**Figure 3:**
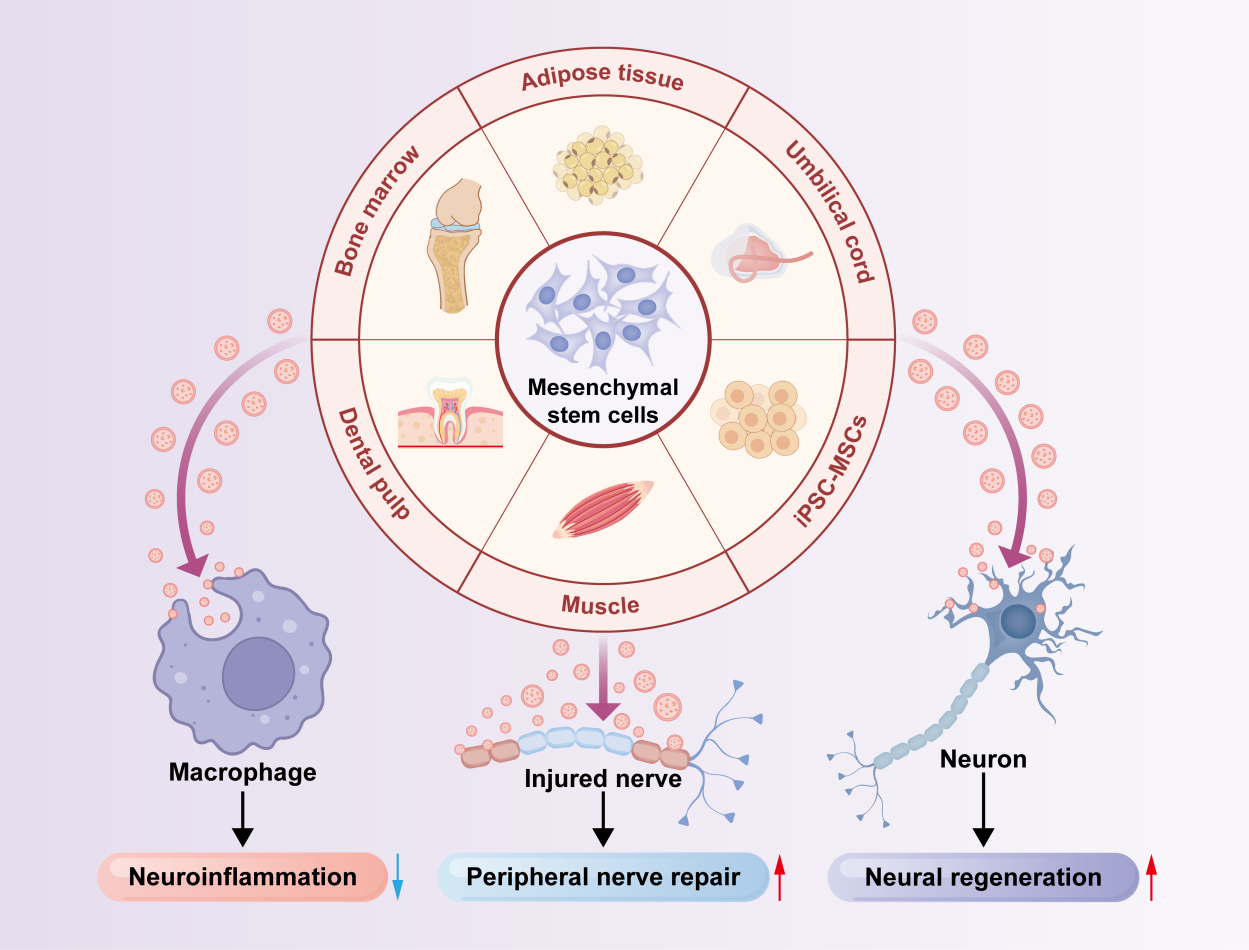
Exosomes derived from various mesenchymal stem cell (MSC) sources, including bone marrow, adipose tissue, umbilical cord, muscle, dental pulp, and induced pluripotent stem cells (iPSCs), were internalized by macrophages and neural cells. These exosomes demonstrate multimodal neuroprotective effects by modulating macrophage polarization, reducing neuroinflammatory responses, enhancing peripheral nerve repair, and promoting nerve regeneration.

Stem cells have become a key tool in the study of neural regeneration due to their potential for self-renewal and multidirectional differentiation. Mesenchymal stem cells (MSCs) are the most widely studied type of stem cells. The advantages of MSCs include a wide range of sources, low immunogenicity, and the ability to differentiate into multiple cell types. The main sources of MSCs include bone marrow, adipose tissue, and human umbilical cord (Gong et al., 2021). A study has shown that bone marrow mesenchymal stem cells (BMSCs) and adipose-derived MSCs have significant effects in repairing CNS injuries by secreting neuroprotective factors, reducing inflammatory responses, and promoting neuronal regeneration (Xue et al., 2024). BMSC-derived exosomes promote peripheral nerve regeneration by regulating the expression of miRNAs, thereby modulating inflammatory responses and neuroprotective pathways (Wang et al., 2024). Additionally, human umbilical cord mesenchymal stem cells (UCMSCs) are considered promising candidate cells for neural repair due to their low immunogenicity and high proliferation capacity. Neural stem cells are also important as they can directly differentiate into neurons and glial cells, making them an ideal cell source for repairing neural injuries (Stenudd et al., 2015). Induced pluripotent stem cell–derived MSCs exosomes activate the brain-derived neurotrophic factor (BDNF)/tropomyosin receptor kinase B (TrkB) signaling pathway and its downstream protein kinase B (Akt), extracellular signal-regulated kinase (ERK), and phospholipase C gamma 1 (PLCγ1)-cyclic adenosine monophosphate response element-binding protein (CREB) pathways by delivering BDNF, thereby improving functional recovery after acute spinal cord injury (Kim et al., 2025). In this context, exosomes secreted by stem cells are gaining increasing attention as an alternative therapeutic strategy to EVs (**Additional Table 2**; Zhou et al., 2020; Chen et al., 2021; Sha et al., 2021; Ma et al., 2022; Liu et al., 2024b; Qin et al., 2024). This innovative application mitigates the risks associated with cell transplantation and enables precise molecular delivery (**Figure 3**).

**Additional Table 2:** Role of exosomes derived from mesenchymal stem cells in neurological diseases

Neurological disorder	Source of exosomes	Cargo	Molecular target	Reference
Brain-related diseases	BMSCs	miR-145	FOXO1	Zhou et al., 2022a
		miR-223-3p	CysLT2R	Zhao et al., 2020a
		miR-148b-3p	DLL4/Notch1	Yi et al., 2024
		miR-23b	PTEN/Nrf2	Hu et al., 2023a
		miR-26a-5p	CDK6	Cheng et al., 2021
	UCMSCs	miR-22-3p	mTORCl pathway	Sang et al., 2023
		HGF, BDNF and TNFR1	MDA and T-SOD	Liu et al., 2024a
	NSCs	miR-150-3p	CASP2	Luo et al., 2022
		miR-128-3p	BMP, OPCs	Hou et al., 2023
Spinal cord-related diseases	BMSCs	miR-26a	PTEN/AKT/mTOR pathway	Chen et al., 2021
		miR-431-3p	RGMA	Sun et al., 2024
		miR-199a-5p	GSK-3β	Yang et al., 2023
		miR-146a	IRAK1/TRAF6/NF**-**κB p65 pathway	Tang et al., 2024
		miR-23b-3p	NEK7/NLRP3	Wang et al., 2023
		miR-367-3p	EZH2	Fan et al., 2023
		lncGm36569	miR-5627-5p/F SP1	Shao et al., 2022
	UCMSCs	miR-501-5p	MLCK	Xie et al., 2023
		VEGF and BFGF	PI3K/Akt/mTOR pathway	Li et al., 2022
		miR-23a-3p	Tbr1/Wnt pathway and PBK/Akt pathway	Qin et al., 2024
		miR-146a-5p	Traf6 and Irak1	Lai et al., 2022
	NSCs	miR-124-3p	MYH9	Jiang et al., 2020
		let-7b-5p	LRIG3	Liu et al., 2024b
Neurodegenerative diseases	UCMSCs	MG53 BDNF	Nrf2 pathway microtubule-associated protein 2	Ma et al., 2022 Zhou et al., 2020
	MSCs	miR-29c-3p	BACE1	Sha et al., 2021
Peripheral nerve regeneration	BMSCs ADMSCs	miR-214-3p BDNF and NGF	MED19 PI3K/AKT pathway and MAPK/ERK pathway	Wang et al., 2024 Bucan et al., 2019
		miR-22-3p	AKT/mTOR pathway	Yang et al., 2022
	UCMSCs	BDNF	PBK/Akt pathway and ERK/MAPK pathway	Zhang et al., 2020a

ADMSCs: Adipose-derived mesenchymal stem cells; BACE1: beta-site amyloid precursor protein cleaving enzyme 1; BMP: bone morphogenetic protein; BMSCs: bone marrow-derived stem cells; BDNF: brain-derived neurotrophic factor; GSK-3β: glycogen synthase kinase 3 beta; HGF: hepatocyte growth factor; MAPK/ERK pathway: mitogen-activated protein kinase/extracellular signal-regulated kinase pathway; mTORCl: mechanistic target of rapamycin complex 1; MSCs: mesenchymal stem cells; MDA: malondialdehyde; NSCs: neural stem cells; OPCs: oligodendrocyte precursor cells; PI3K/Akt pathway: phosphoinositide 3-kinase/protein kinase B pathway; PTEN/AKT/mTOR pathway: phosphatase and tensin homolog/protein kinase b/mechanistic target of rapamycin pathway; RGMA: repulsive guidance molecule A; T-SOD: total superoxide dismutase; TNFR1: tumor necrosis factor receptor 1.

#### Bone marrow mesenchymal stem cells

With the development of stem cell therapy, an increasing number of studies have reported that BMSC-derived exosomes have important biological functions in nerve repair. BMSC have become an ideal candidate cell source for nerve regeneration therapy due to their high proliferation ability, immunomodulatory function, and low immunogenicity (Hu et al., 2023b; Li and et al., 2024). First, BMSC-derived exosomes play a key role in immunoregulation. A study has shown that BMSC-derived exosomes inhibit local inflammatory responses following nerve injury by promoting the polarization of M2 macrophages, creating a favorable microenvironment for nerve repair and reducing cell apoptosis (Xue et al., 2024). This process further confirms the potential of BMSC-derived exosomes as an effective immunomodulatory tool in nerve regeneration. Second, BMSC-derived exosomes enhance the overall effect of nerve repair by promoting the proliferation and migration of neurons and neural supporting cells (such as Schwann cells and glial cells). For example, specific miRNAs in exosomes, such as miR-214-3p, can directly regulate the function of Schwann cells by targeting MED19, thereby enhancing their repair effect following nerve injury (Hu et al., 2023b). This mechanism promotes cell proliferation and migration during nerve repair by regulating intracellular signaling pathways, such as the PI3K/Akt pathway.

In addition, BMSC-derived exosomes promote axon regeneration and functional recovery after spinal cord injury. Single-cell RNA sequencing revealed that BMSC-derived exosomes from specific subpopulations (such as CD271^+^CD56^+^) can significantly enhance the nerve regeneration effect in a spinal cord injury model (Sun et al., 2024). These exosomes facilitate the repair of the injured site and the recovery of nerve function by promoting axon growth and the interaction between neurons and Schwann cells. Regarding the molecular mechanism, BMSC-derived exosomes have been shown to enhance the proliferation and migration of Schwann cells by regulating the circRNA-Nkd2 and the miR-214-3p/MED19 axis, thereby promoting the repair of facial nerve injury. This process not only accelerates nerve repair but also reduces the formation of glial scars, a common occurrence during nerve repair, by regulating TGF-β signaling, further improving the regenerative effect (Madduri et al., 2024). BMSC-derived exosomes carry miR-23b, which primarily reduces oxidative stress and neuroinflammation after intracerebral hemorrhage by regulating the PTEN/Nrf2 antioxidant pathway and the PTEN/NLRP3 inflammasome pathway (Hu et al., 2023a).

BMSC-derived exosomes have shown significant neuroprotective and regenerative potential in brain-related diseases, such as ischemic stroke and TBI. One study found that miR-26a-5p, delivered by MSC-derived exosomes, can inhibit CDK6. This target can block CDK6-mediated G1/S phase transition and apoptosis-related signals, thereby reducing microglial apoptosis and alleviating cerebral I/R injury (Cheng et al., 2021). Engineered 293F cell-derived exosomes eliminate p21^+^CD86^+^ microglia through targeted delivery of quercetin, inhibit the p21–C/EBPβ-dependent SASP pathway, thereby alleviating neuroinflammation, protecting the CNS, and promoting recovery from ischemic stroke (Yang et al., 2025). A study showed that miR-223-3p can also inhibit microglial M1 polarization and promote M2 transformation, and it exerts anti-inflammatory and neuroprotective effects by targeting and inhibiting CysLT2R (Zhao et al., 2020a). A study confirmed that BMS exosomes specifically inhibit the DLL4/Notch1 pathway by delivering miR-148b-3p, thereby blocking the activation of microglia and inflammatory response, which provides a new mechanism for alleviating cerebral I/R injury (Yi et al., 2024).

BMSC-derived exosomes have been reported to have significant therapeutic effects in spinal cord injury repair and the reconstruction of neural function through a multi-target mechanism of action. First, these exosomes excel in regulating the inflammatory microenvironment, effectively promoting tissue repair by bidirectionally modulating the expression of inflammatory factors. Specifically, BMSC-derived exosomes significantly downregulate pro-inflammatory factors such as tumor necrosis factor-alpha and interleukin-1beta (IL-1β) while upregulating levels of anti-inflammatory mediators such as interleukin-10 (IL-10). This bidirectional regulatory capacity enables them to effectively reshape the immune microenvironment in the injured area (Ye et al., 2024). Second, the literature indicates that BMSC-derived exosomes can accurately regulate the proliferation and differentiation of neural stem cells by delivering miR-199a-5p, which significantly enhances the reconstruction of damaged neural function (Yang et al., 2023). The protective effects of BMSC-derived exosomes on nerve cells are also evident in their excellent anti-apoptotic and anti-oxidative stress capabilities. One study demonstrated that by intervening in key signaling pathways, such as IRAK1/TRAF6/NF-κB p65, BMSC-derived exosomes can effectively block inflammatory responses and inhibit oxidative stress damage, thus providing multiple protective barriers for nerve cells (Tang et al., 2024). Moreover, BMSC-derived exosomes, through the delivery of miR-23b-3p, specifically inhibit the NEK7/NLRP3 inflammasome pathway. This inhibitory effect blocks microglial pyroptosis, significantly alleviating the inflammatory response and demyelinating pathology in experimental autoimmune encephalomyelitis (Wang et al., 2023). A study also showed that miR-367-3p alleviates autoimmune encephalomyelitis by inhibiting EZH2 and upregulating the SLC7A11/GPX4 pathway, thereby preventing microglial cell ferroptosis (Fan et al., 2023). Recent systematic reviews and meta-analyses have provided robust evidence for the therapeutic potential of BMSC-derived exosomes. One study found that these exosomes significantly improve motor function in rats with spinal cord injury by regulating the inflammatory response, reducing cell apoptosis, and promoting axon regeneration. Another recent study revealed that BMSC-derived exosomes intervene in cell signal transduction through the synergistic effects of long non-coding RNAs and microRNAs, offering a new perspective for their application in spinal cord injury repair. Additionally, a study confirmed that BMSC-derived exosomes effectively inhibit ferroptosis in neurons via the lncGm36569/miR-5627-5p/FSP1 molecular regulatory axis, significantly improving neurological deficits caused by spinal cord injury (Shao et al., 2022). In summary, BMSC-derived exosomes, with their multi-target and multi-dimensional action characteristics, show promising clinical application prospects in the field of spinal cord injury repair. Existing evidence provides a crucial theoretical basis for developing novel neural repair strategies through synergistic mechanisms, such as regulating the inflammatory microenvironment, promoting nerve regeneration, and inhibiting cell death (Shang et al., 2024; An et al., 2025).

#### Adipose-derived mesenchymal stem cells

Adipose-derived MSCs are multipotent stem cells that are abundant and easy to obtain. The exosomes they secrete have demonstrated potential for application in nerve regeneration and repair (Qin et al., 2023). Adipose-derived MSC exosomes work synergistically to enhance nerve repair through various molecular mechanisms.

First, these exosomes are rich in neurotrophic factor-related transcripts, such as BDNF and nerve growth factor (NGF), which can activate signaling pathways in damaged nerve cells, including the PI3K/AKT and MAPK/ERK pathways. These pathways promote nerve cell survival and enhance axon growth. *In vitro* experiments have shown that adipose-derived MSC exosomes significantly promote neurite extension. Additionally, in animal models of sciatic nerve injury, these exosomes enhanced nerve regeneration at the injured site (Bucan et al., 2019). Second, adipose-derived MSC exosomes optimize the neural regeneration microenvironment by regulating the function of Schwann cells. One study demonstrated that these exosomes can be internalized by Schwann cells, promoting their proliferation, migration, and myelination. This mechanism relies on the activation of the ERK1/2 and STAT3 pathways by miRNAs and proteins present in the exosomes, which upregulate the expression of myelin proteins P0 and MBP, thereby enhancing myelination (Chen et al., 2019). Furthermore, miRNA-22-3p in exosomes has been shown to downregulate PTEN expression, activate the AKT/mTOR pathway, and further promote the proliferation and migration of Schwann cells, supporting neural regeneration (Yang et al., 2022). Adipose-derived MSC exosomes also indirectly promote neural regeneration by regulating inflammatory and apoptotic mechanisms. One study found that anti-inflammatory miRNAs (such as miR-146a) and antioxidant enzymes (such as SOD1) present in exosomes can inhibit the NF-κB signaling pathway, reducing the inflammatory response at the injury site and mitigating oxidative damage to nerve cells (Fang et al., 2019). Additionally, a recent study showed that angiogenic factors (such as vascular endothelial growth factor and stromall cell-derived factor 1) in exosomes significantly promote angiogenesis, providing oxygen and nutritional support to the injured site, thereby further enhancing nerve regeneration (Xiong et al., 2020). In summary, adipose-derived MSC exosomes exhibit great potential in nerve regeneration and repair through mechanisms such as neurotrophic factor regulation, anti-inflammatory and antioxidant protection, and angiogenesis.

Adipose-derived MSCs (ADMSCs) have shown significant clinical application prospects in the treatment of brain diseases, such as cerebrovascular diseases, brain tumors, and brain trauma. Based on their multi-directional differentiation potential and immunomodulatory properties, ADMSCs have become a promising cell therapy strategy in the field of neurological diseases (Mattei and Delle Monache, 2024; Zhang et al., 2024a). Under the pathological conditions of cerebrovascular diseases, ADMSCs significantly improve the process of neurological function repair after ischemic brain injury by inducing angiogenesis and neural regeneration mechanisms. One study has found that ADMSCs can secrete a variety of neurotrophic factors; BDNF binds to the TrkB receptor, and nerve growth factor acts on the TrkA receptor to activate the PI3K/Akt signaling pathway, reducing the expression of apoptosis-related factors, and thereby reducing the apoptosis rate and protecting damaged neurons (Bagheri-Mohammadi, 2021; Ejma et al., 2022).

#### Umbilical cord mesenchymal stem cells

Umbilical cord mesenchymal stem cell (UCMSC)-derived exosomes have shown great application potential in the field of neural regeneration and repair, with in-depth analyses of their mechanism of action. These exosomes promote the survival, differentiation, and functional recovery of nerve cells by carrying bioactive molecules and activating cell signaling pathways (Xie et al., 2023). One study has found that UCMSC-derived exosomes significantly improve motor and sensory functions in the repair of sciatic nerve injury. This effect is mainly achieved by enhancing the expression of BDNF and activating the PI3K/Akt and ERK/MAPK pathways, thereby promoting neuronal growth and survival (Zhang et al., 2020a). In addition, when UCMSC-derived exosomes were pretreated with hypoxia, they were shown to carry more angiogenic factors (such as vascular endothelial growth factor and basic fibroblast growth factor), resulting in activation of the PI3K/Akt/mTOR signaling pathway in endothelial cells, promoting the formation of new blood vessels, improving the blood supply to local tissues, and provide support for neural regeneration in a model of spinal cord injury (Li et al., 2022). UCMSC-derived exosomes have been shown to activate the mTORC1 signaling pathway through the transport of microRNAs (such as miR-22-3p), which can repair optic nerve damage. mTORC1 promotes axon regeneration and the survival of retinal ganglion cells by regulating protein synthesis and cell growth-related genes, while inhibiting apoptosis signaling pathways (Sang et al., 2023).

Exosomes derived from umbilical cord–derived neural stem cells (UCNSCs) have demonstrated significant clinical therapeutic effects in the treatment of craniocerebral trauma. A recent study revealed that these exosomes significantly inhibit post-traumatic neuroinflammatory responses by regulating the activation states of microglia and astrocytes, thereby improving neurological function recovery (Cui et al., 2022). Notably, these exosomes effectively suppress the programmed cell death pathway by activating the mitochondrial autophagy mechanism mediated by the PINK1/Parkin signaling pathway, exhibiting remarkable neuroprotective properties in models of traumatic brain injury (TBI) (Zhang et al., 2023). Further research has shown that UCNSC-derived exosomes have significant therapeutic effects in animal models of cerebral hemorrhage and ischemic brain injury. Mechanistic studies confirmed that these exosomes regulate the levels of malondialdehyde and total superoxide dismutase through the synergistic effects of multiple factors, including hepatocyte growth factor, BDNF, and TNFR1. This regulation effectively alleviates inflammatory responses and oxidative stress damage, reducing brain tissue injury caused by brain trauma (Liu et al., 2024a). Additionally, molecular mechanism studies have identified specific microRNAs, such as miR-145, carried by exosomes that reduce the rate of cell apoptosis in ischemia–reperfusion injury by targeting and regulating FOXO1 gene expression, thereby producing a neuroprotective effect (Zhou et al., 2022a). Together, these studies provide a systematic explanation of the action network of UCMSC-derived exosomes in the treatment of neurological diseases, establishing a solid theoretical foundation and clinical application potential. Exosomes derived from human UCMSCs also exhibit significant anti-inflammatory properties and neuroregenerative potential in the treatment of spinal cord injury (Sun et al., 2023).

Mechanistic studies have shown that these exosomes significantly inhibit the expression of pro-inflammatory factors, such as tumor necrosis factor-α and interleukin-6, effectively alleviating the neuroinflammatory microenvironment and promoting the dual repair of damaged neural tissue structure and function (Yang et al., 2024b; Zhang, 2024). Exosomes-derived from human UCMSCs target ZMYND8, CTSC, SPINT1, CLMN, and AKAP12 by delivering cargo such as miR-27a-3p, miR-132-3p, miR-92a-3p, miR-181b-3p, and miR-210-3p. This cargo promotes neurogenesis, synaptic remodeling, and angiogenesis, enhancing neuroplasticity and functional recovery following a stroke (Kim et al., 2025). Importantly, exosomes-derived from human UCMSCs play a crucial regulatory role in the directional differentiation of neuronal lineages and the maturation of oligodendrocytes. Experimental data have demonstrated that these exosomes activate neuronal regeneration programs and improve nerve conduction function through miR-146a-5p (Lai et al., 2022). This miRNA-mediated epigenetic regulatory mechanism significantly enhances the biological activity of exosomes, producing a synergistic therapeutic effect on neuroprotection and regeneration. In-depth studies have shown that exosomes-derived from human UCMSCs provide microenvironmental support for injury repair by promoting angiogenesis and improving the structural integrity of the blood–spinal cord barrier (Divband et al., 2022; Li et al., 2025). These exosomes regulate the stability of YAP protein by intervening in the CK1δ/β-TRCP signaling axis, effectively inhibiting post-injury fibrosis and inflammatory cascades, ultimately achieving neural function remodeling (Ji et al., 2020). A study on Alzheimer’s disease has shown that UCMSC-derived exosomes significantly delay the pathological process of neurodegeneration through a dual mechanism of immunomodulation and anti-inflammation. Collectively, these research findings systematically construct a molecular framework for understanding how exosomes can be utilized to treat neurological diseases, paving the way for new clinical translational applications.

#### Neural stem cells

Neural stem cells (NSCs) have shown great potential in the regeneration and repair of the nervous system due to their ability to self-renew and differentiate into neurons and glial cells (Liu et al., 2023). These cells directly participate in the structural restoration of damaged neural tissue and regulate the neural microenvironment by secreting various bioactive molecules, thus playing a key role in the repair of CNS damage (Kawai et al., 2021). One study has confirmed that NSCs can effectively reduce neuroinflammatory responses, promote neuronal survival, and enhance neurological function recovery, providing new therapeutic ideas for the treatment of stroke, brain trauma, and other neurodegenerative diseases (Li et al., 2023).

In recent years, neural stem cell-derived exosomes have been considered to be an important mediator of the role of NSCs in the process of neural repair. Compared to direct transplantation of NSCs, exosomes have lower immunogenicity and a higher safety profile; therefore, they are widely considered a more ideal treatment strategy. The miRNAs enriched in NSCs play an important role in regulating neuronal apoptosis, promoting neuronal proliferation, and regulating myelin repair. One study has found that miR-150-3p significantly reduces neuronal apoptosis and enhances neuronal proliferation by targeting caspase-2. In a model of hypoxic–ischemic brain injury, NSC-derived exosomes transporting miR-150-3p not only reduced the volume of cerebral infarction but also significantly improved neurological function recovery (Luo et al., 2022). In addition, miR-128-3p in NSC-derived exosomes reversed the inhibition of oligodendrocyte precursor cell (OPC) differentiation caused by fibrinogen accumulation and promoted remyelination by preventing fibrinogen-induced demyelination associated with the bone morphogenetic protein signaling pathway. This mechanism of action has been validated as an effective repair strategy in a cerebral ischemia model (Hou et al., 2023).

One study has confirmed that NSC-derived exosomes can significantly inhibit the abnormal activation of neurotoxic microglia by delivering miR-124-3p and effectively regulate the overactivation of astrocytes, thereby accelerating the recovery of motor function in models of spinal cord injury (Jiang et al., 2020). A previous study has shown the neuroprotective effect of NSC-derived exosomes; these exosomes can significantly improve the survival of NSCs in hypoxic microenvironments, which effectively maintain their normal physiological functions by targeted delivery of specific miRNA molecules, such as miR-210-3p (Li et al., 2020). One study has also found that exosomes can effectively block the ferroptosis process of nerve cells by regulating key nodes of ferroptosis-related signaling pathways, creating favorable conditions for neural function remodeling (Shao et al., 2022).

### Neural cell–derived exosomes

The complexity of the nervous system determines the diversity of its constituent cells, which play a vital role in nerve regeneration and repair. Neurons are the core unit responsible for signal transmission in the nervous system (Ransohoff, 2016). In addition, microglia and astrocytes in the CNS and Schwann cells in the peripheral nervous system play an indispensable role in maintaining neural homeostasis and injury repair. Microglia, as immune cells of the CNS, promote post-injury repair by clearing diseased tissue and releasing neurotrophic factors. Astrocytes not only provide metabolic support for neurons, but also participate in the formation of reparative glial scars when nerves are injured (Allen and Lyons, 2018). In the peripheral nervous system, Schwann cells promote axon regeneration through the formation of myelin and the secretion of neurotrophic factors. The use of exosomes secreted by these cells has been shown to be more efficient and safer than direct cell transplantation (Hastings and Valdez, 2024). Therefore, exploring how nerve cells regulate the process of nerve regeneration and repair through exosomes has become a field with high research potential.

**Figure 4:**
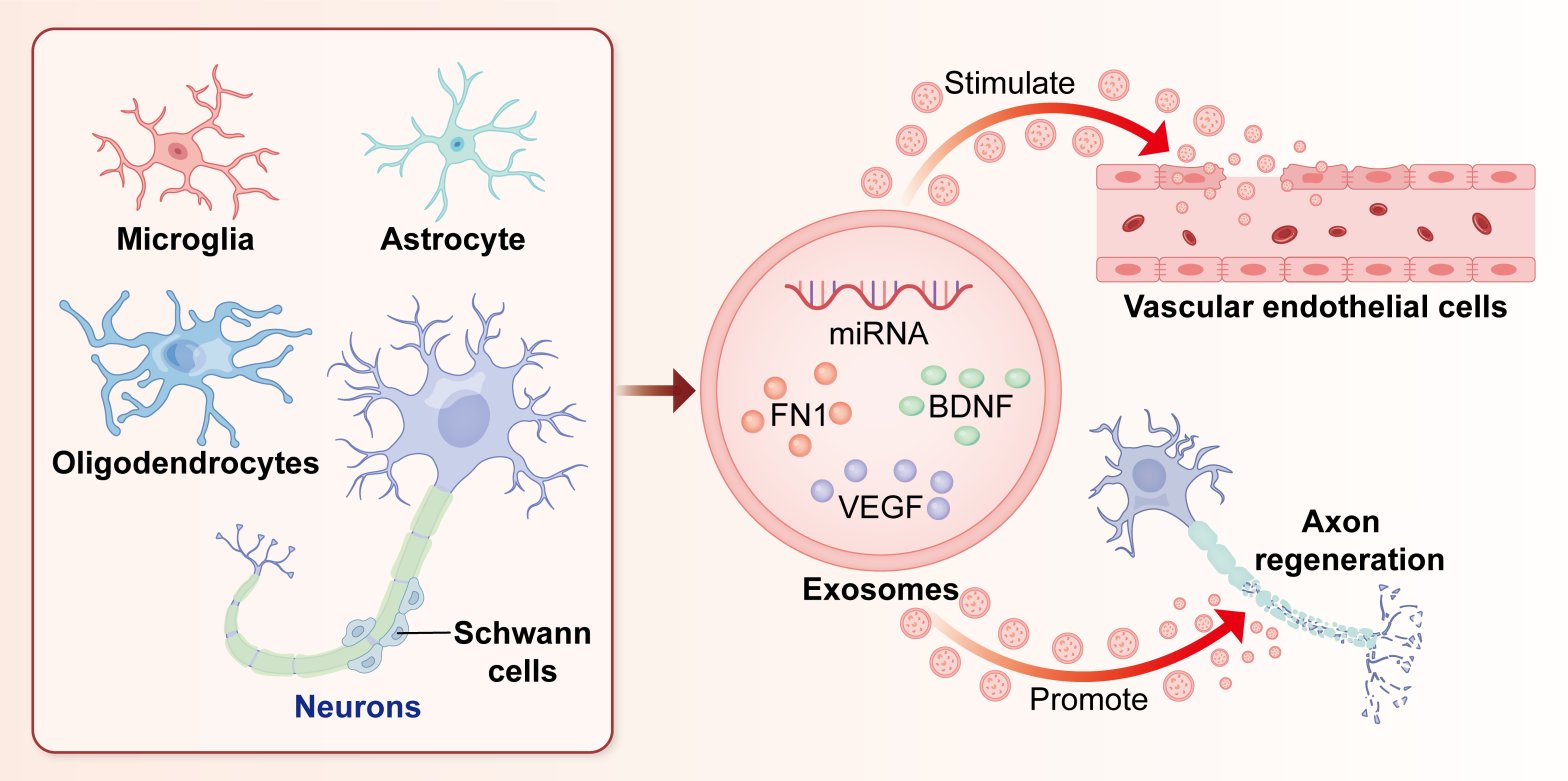
Exosomes secreted by microglia, astrocytes, neurons, Schwann cells, and oligodendrocytes promote vascular endothelial cell regeneration and facilitate neural repair. This process occurs through the targeted delivery of bioactive substances, including microRNA, BDNF, and VEGF. These findings suggest a potential therapeutic strategy for treating neurological injuries and diseases. BDNF: Brain-derived neurotrophic factor; VEGF: vascular endothelial growth factor.

Exosomes derived from neural cells play a crucial role in neural regeneration and protection in brain diseases, spinal cord lesions, and neurodegenerative disorders (**Figure 4**). One study has shown that neuronal exosomes can effectively reverse the overactivation state of microglia, regulate the phenotypic transformation of astrocytes, promote the maturation and differentiation of OPCs, and significantly improve the differentiation efficiency of neural stem cells into functional neurons, ultimately achieving accelerated repair in spinal cord injury (Xu et al., 2023b; Zhu et al., 2024). In the field of brain disease treatment, the application prospects of exosomes are also worthy of attention. Their unique ability to penetrate the BBB makes exosomes an ideal biological carrier, which can achieve targeted delivery of therapeutic molecules in the CNS and provide new strategies for the intervention of neurological diseases, such as AD and PD (Fan et al., 2022; Shetgaonkar et al., 2022). In summary, neurogenic exosomes have dual value in the field of neural repair; they can be used as biomarkers for early diagnosis and as therapeutic carriers, opening up innovative ways for the precise diagnosis and treatment of neurological diseases (Xia et al., 2019).

#### Microglia

Microglia are the primary resident immune cells in the CNS (Borst et al., 2021). They originate from primitive myeloid cells of the embryonic yolk sac and have remarkable plasticity. Microglia are divided into two main phenotypes: the pro-inflammatory phenotype (M1 type) and the anti-inflammatory phenotype (M2 type). M1 microglia participate in inflammatory responses by releasing pro-inflammatory factors, which may aggravate nerve damage. In contrast, M2 microglia play an important role in neuroprotection, axon regeneration, and tissue repair by secreting anti-inflammatory factors and neurotrophic factors (Guo et al., 2021). In recent years, it has been found that microglia not only affect nerve regeneration through direct cell-to-cell interactions but also regulate the neural microenvironment by secreting exosomes, a key extracellular vesicle, thereby promoting neural repair.

Exosomes secreted by microglia are nanoscale membrane-bound vesicles, which have high stability and good ability to cross the BBB. Exosomes derived from M2 microglia can regulate gene expression in recipient cells by delivering specific miRNAs, such as miR-124, miR-151-3p, and miR-672-5p, thereby promoting neural repair (Song et al., 2019; Li et al., 2021). A study has found that miR-124 carried by exosomes derived from M2 microglia can inhibit cell apoptosis and promote neuronal survival by downregulating USP14, thereby alleviating ischemic brain injury (Song et al., 2019). Furthermore, it has been reported that miR-151-3p reduces neuronal apoptosis and promotes axonal regeneration by regulating the p53/p21/CDK1 signaling pathway, thereby improving functional recovery following spinal cord injury (Li et al., 2021). One study found that miR-672-5p played a key role in the repair of traumatic spinal cord injury by inhibiting the AIM2/ASC/C ASP1 signaling pathway and reducing neuronal pyroptosis (Zhou et al., 2022b). Microglial exosomes can also promote angiogenesis and tissue repair through antioxidant mechanisms. One study has shown that key molecules in exosomes promote neural regeneration by activating the Keap1/Nrf2/HO-1 signaling pathway, reducing the accumulation of reactive oxygen species, and improving oxidative stress in the microenvironment (Peng et al., 2021). In summary, microglial exosomes play a crucial role in regulating inflammation, anti-apoptosis, and neural regeneration by carrying specific bioactive molecules, providing a significant research basis for the treatment of neural injury.

As resident immune cells of the CNS, microglia play dual regulatory roles in neuroinflammation and neuroprotection. Recent evidence suggests that microglia-derived exosomes possess significant neuroregenerative potential and protective properties in the context of brain-related diseases. The primary research focus has been on the mechanisms by which microglia-derived exosomes act in cerebral ischemia. Experimental data indicate that microglia regulate neuroinflammation through the secretion of exosomes. The microRNAs and specific functional proteins contained within these exosomes can effectively promote neuronal survival and functional recovery. Notably, microglia-derived exosomes exhibit significant neuroregenerative effects during the secondary injury phase following cerebral hemorrhage. These nanoscale vesicles help maintain the homeostasis of the BBB and promote angiogenesis through a dual regulatory mechanism, thereby significantly improving the brain microenvironment after injury and providing structural support for neuroregeneration (Gao et al., 2020b; Kang et al., 2020). As the academic community increasingly recognizes the dynamic regulatory functions of microglial exosomes in the pathological contexts of stroke and brain injury, their potential therapeutic applications continue to gain attention.

As a core regulator of the microenvironment following spinal cord injury, microglia play a key pathophysiological role (Wan et al., 2022). Microglia-derived exosomes have been reported to inhibit the neuroinflammatory cascade and antagonize programmed cell death through a dual regulatory mode, significantly promoting the process of post-injury neural regeneration. Typical examples show that miRNA molecules in exosomes precisely regulate the phenotypic polarization of microglia, driving them to transform into the M2 anti-inflammatory phenotype with neuroprotective properties, effectively blocking inflammatory signal transduction and activating regeneration pathways. One study has shown that specific miRNA components (such as miR-383-3p) precisely regulate the process of neuronal programmed cell death by targeting and inhibiting the ATF4 signaling axis, providing new insights into maintaining neuronal homeostasis (Wei et al., 2021). Furthermore, miR-5121 has been shown to effectively relieve growth inhibition signals by directly targeting RGMa, which significantly enhances synaptic remodeling efficiency and accelerates neural function compensation (Zhao et al., 2021a).

Current studies have shown that the mechanism of action of microglia-derived exosomes in neurodegenerative diseases primarily involves the following aspects (Gupta and Mazumder, 2022; Rasouli et al., 2024). The primary pathway of action is that the various anti-inflammatory mediators and neurotrophic factors they carry can effectively inhibit the neuroinflammatory cascade and mediate the process of neuroregeneration. Rasouli et al. (2024) have confirmed that the specific miRNA molecules contained in them can precisely regulate the gene expression network of neural cells through epigenetic regulatory mechanisms, thereby regulating the survival and function of neurons. It is worth noting that these nanovesicles can effectively remove abnormal protein aggregation and maintain neuronal homeostasis by regulating the key nodes of the autophagy–lysosome pathway and apoptosis-related signals. In the pathological process of neurodegenerative diseases, such as AD and PD, the bidirectional regulatory characteristics of microglial exosomes have become a promising therapeutic target. By precisely regulating the M1/M2 phenotype conversion of microglia and their exosome secretion pattern, the neuroinflammatory microenvironment can be significantly improved, while activating the endogenous neuroregeneration mechanism (Gupta and Mazumder, 2022).

#### Astrocytes

Astrocytes are one of the most important types of glial cells in the CNS. Their number far exceeds that of neurons, and they play a crucial role as important regulators of neural tissue homeostasis. Astrocytes function to provide metabolic support for neurons, regulate ion balance, and participate in the formation of synapses and signal transduction (Giovannoni and Quintana, 2020). Following nerve injury, astrocytes show a high degree of dynamic responsiveness through activation. Activated astrocytes are divided into type A1 and type A2; type A1 astrocytes are associated with pro-inflammation and neurotoxicity, while type A2 promotes neuroprotection and repair by secreting neurotrophic factors (Chen et al., 2020). In addition, astrocytes participate in post-injury nerve regeneration and axon repair by regulating the permeability of the BBB, clearing metabolic waste from the injured site, and secreting exosomes. One study has shown that exosomes secreted by astrocytes play an important role in the repair of cerebral ischemia, TBI, and degenerative diseases of the CNS (Wu et al., 2020).

Zhang et al. (2021b) have found that exosomes secreted from astrocytes are highly stable and targeted, making them key mediators of nervous system signaling. Exosomes secreted by astrocytes are rich in microRNA-34c, which can target TLR7, regulate the NF-κB and MAPK signaling pathways, reduce inflammatory responses and neuronal apoptosis, and thus alleviate cerebral ischemia–reperfusion injury (Wu et al., 2020). In addition, astrocyte exosomes also improve the mitochondrial function of damaged neurons by delivering the gap junction protein fragment GJA1-20k. GJA1-20k plays a key role in neural repair after TBI by downregulating the expression of the apoptosis-related proteins BAX and C ASP3, enhancing mitochondrial metabolism and ATP production (Chen et al., 2020). Another study showed that exosomes secreted by astrocytes under hypoxia and glucose deprivation can significantly promote the migration and differentiation of OPCs. During this process, exosomes deliver specific miRNAs and proteins, among which miR-219 and miR-338 are considered to be key regulators of oligodendrocyte differentiation. They promote the differentiation of oligodendrocytes into mature types by inhibiting the expression of target genes (*PDGFRα* and *SOX6*) associated with differentiation inhibition. In addition, exosomes carry neurotrophic factors, including BDNF and insulin-like growth factor 1, which support the migration and differentiation of oligodendrocytes. Its mechanism of action involves the activation of downstream PI3K/Akt and MAPK/ERK signaling pathways, which regulate the expression of genes related to cell survival, proliferation, and differentiation, and play a significant role in the repair of ischemic brain injury (Xu et al., 2021).

As the most abundant glial cell in the CNS, astrocytes participate in neuroregeneration and neuroprotection by secreting exosomes and have irreplaceable biological functions in maintaining neural homeostasis. Under pathological conditions, such as stroke, astrocyte-derived exosomes reshape the brain microenvironment by regulating inflammatory responses, antagonizing oxidative stress, and promoting angiogenesis and axon extension through multiple pathways. This process has been shown to significantly reduce the incidence of neuronal degeneration and accelerate the reconstruction of neurological function (Xie et al., 2022). One study has confirmed that astrocyte-derived exosomes have an apparent neuroprotective effect on cerebral ischemia–reperfusion injury. One study has shown that miR-29a enriched in exosomes can effectively alleviate oxidative stress damage and inhibit inflammatory responses by targeting and inhibiting TP53INP1 and regulating the NF-κB/NLRP3 signaling pathway (Liu et al., 2022a). It is worth noting that this type of exosome can finely regulate neuronal plasticity through the miRNA-mediated gene expression regulatory network, providing a molecular basis for neuroregeneration and repair (Bai et al., 2021). In the pathological process of brain tumors, astrocyte-derived exosomes have been shown to participate in the reprogramming process of the tumor microenvironment and perform key pathophysiological functions by bidirectionally regulating the tumor-promoting/tumor-suppressing signaling network. Therefore, existing research evidence demonstrates that astrocyte-derived exosomes show bidirectional regulatory characteristics in the field of neural regeneration and protection (Zhao et al., 2021b).

As important regulators of CNS homeostasis, astrocytes perform key physiological functions through paracrine exosomes, and their mediated neuroregeneration and neuroprotective effects have received extensive academic attention. In the pathological process of spinal cord injury, astrocyte-derived exosomes have been shown to mediate the process of neuroremodeling and promote the repair of the post-injury microenvironment through spatiotemporal-specific regulatory mechanisms. These exosomes coordinate neuroinflammatory homeostasis through a dual mechanism, which can effectively improve motor function prognosis and reduce the risk of secondary neurological injury (Cai et al., 2024). It is worth noting that astrocyte-derived exosomes show clinical translational prospects in intervention strategies for neurodegenerative diseases, such as AD. These nanovesicles not only carry disease-specific molecular tags but also implement precise regulation through bidirectional regulation of neuroinflammatory pathways and neuroprotective signaling networks, providing a molecular basis for the development of new diagnostic and therapeutic strategies. One study has revealed that regulatory factors, such as miR-132 carried by exosomes, can effectively curb inflammation-related neural damage in the process of neurodegenerative lesions by regulating the activation state of microglia temporally. It is particularly noteworthy that this type of exosome demonstrates unique biological value in the field of neural regeneration by regulating the fate determination mechanism of NSCs. Experimental data have shown that the bioactive components carried by exosomes can activate the self-renewal program of NSCs and guide their directional differentiation, thereby systematically constructing a neural repair microenvironment (Ashique et al., 2024). Therefore, existing research has demonstrated that astrocyte-derived exosomes construct a complex neural regeneration and protection network through multidimensional regulatory characteristics, such as intercellular information transmission, neuroimmune regulation, and stem cell fate programming. These groundbreaking discoveries have not only revolutionized our understanding of the repair mechanism of spinal cord injury but have also laid an innovative theoretical framework and translational medicine path for the development of precision medicine solutions based on exosomes.

#### Schwann cells

Schwann cells are the dominant glial cells in the peripheral nervous system. Their main functions include forming myelin, supporting axons, and maintaining neuronal survival. During myelination, Schwann cells wrap around axons multiple times to create a tight membranous structure, which significantly improves the efficiency of nerve signal conduction. In addition, non-myelinating Schwann cells also play an important role in maintaining metabolic support for small-diameter axons and providing neuroprotection (Wei et al., 2024). In the event of nerve injury, Schwann cells show significant responsiveness and can rapidly dedifferentiate into a repair phenotype, followed by proliferation and redifferentiation into myelin-forming cells. This process involves extensive changes in intracellular gene expression, including the activation of c-Jun, which enhances the secretion of neurotrophic factors and the remodeling capacity of the extracellular matrix. The unique axon guidance ability of Schwann cells, along with the secretion of matrix molecules such as laminin and fibronectin, provides important support for axon regeneration in the damaged repair area (Salzer et al., 2024).

The role of Schwann cells in nerve regeneration not only relies on their direct dedifferentiation, proliferation, and redifferentiation functions, but also plays a key role in regulating the injury microenvironment and promoting intercellular signaling by secreting nanoscale EVs, such as exosomes. A study has shown that the delivery of SOX2 and fibronectin 1 by Schwann cells-derived exosomes can activate the PI3K/Akt and MAPK/ERK pathways, respectively, by upregulating cyclin D1 and regulating integrin and actin reorganization, promoting cell proliferation and migration, and providing a molecular basis for the repair of sciatic nerve injury. In addition, Schwann cells–derived exosomes are rich in miR-21, which significantly enhances the activity of the Ras/MAPK pathway by inhibiting SPRY2 (a negative regulator of the Ras/MAPK signaling pathway), thereby accelerating axon growth and neuronal survival. miR-21 can also reduce the inflammatory response at the site of nerve injury, thereby providing a more favorable microenvironment for axon regeneration (Liu et al., 2022b). One study has shown that these exosomes carry a variety of angiogenesis-related factors, such as VEGF, integrin β1, and matrix metalloproteinases. These factors can stimulate the proliferation of vascular endothelial cells and the formation of new capillaries by activating the vascular endothelial growth factor receptor signaling pathway and facilitating integrin-mediated adhesion and migration (Huang et al., 2022). Additionally, matrix metalloproteinases improve the cell migration environment by degrading the extracellular matrix, thereby further accelerating tissue repair.

The neuroregenerative and protective effects of Schwann cells -derived exosomes in the field of spinal cord diseases have become a current research hotspot. In the pathological process of spinal cord injury, Schwann cells-derived exosomes perform core regulatory functions through a variety of mechanisms and play an important role in the process of neuroregeneration. Experimental data show that these exosomes significantly improve the motor function prognosis and reduce the risk of secondary neurological injury by coordinating the dynamic balance between neuroinflammatory homeostasis and promoting neuronal survival and growth. One study has revealed that effector molecules, such as MFG-E8, carried by exosomes regulate the SOCS3/STAT3 signal transduction network through spatiotemporal specificity, accurately guiding the phenotypic transformation of myeloid cells and driving neuroregeneration while inhibiting excessive inflammatory responses (Ren et al., 2023). Notably, this type of exosome plays a crucial role in establishing a neuroprotective microenvironment by mediating transcellular signal cascade reactions. A study has found that miRNAs and signaling proteins carried by exosomes can synergistically regulate the fate determination of neural precursor cells, providing a molecular basis for neuronal survival and the reconstruction of synaptic plasticity. In the pathological microenvironment of diabetes, exosomes secreted by Schwann cells under high glucose stress regulate the expression of target proteins, acting like molecular switches to finely regulate neuronal metabolic homeostasis and functional reconstruction (Jia et al., 2018). The latest research has expanded the cognitive boundaries of this field, revealing that Schwann cells-derived exosomes perform dual regulatory functions in diabetic peripheral neuropathy by regulating the neurovascular unit dialogue mechanism. Mechanistic analysis has shown that exosomes induced by the high glucose microenvironment implement bidirectional regulation through the miR-15b-5p/Txnip molecular axis, which not only antagonizes oxidative damage but also coordinates the regeneration process of the nerve–bone interface (Wang et al., 2025). Based on the existing evidence, Schwann cells-derived exosomes construct a complex neural repair system through multidimensional regulatory characteristics (Huang et al., 2021; Pan et al., 2021; Chen et al., 2022, 2023a,b; Jiang et al., 2024), such as inflammatory network reprogramming, cell communication decoding, and redox homeostasis maintenance (**Additional Table 3**).

**Additional Table 3:** Role of nerve cell-derived exosomes in neurological diseases

Neurological disorder	Source of exosomes	Cargo	Molecular target	Reference
**Brain-related diseases**	Microglia	miR-124 miR-383-3p	USP14 gene ATF4	Song et al., 2019 Wei et al., 2021
		miR-5121	RGMa	Zhao et al., 2021a
		miR-155-5p	Socsl and NF-κB	Chen et al., 2023b
	Astrocytes	GJA1-20k	Bax and caspase-3	Chen et al., 2020
		miR-34c	TLR7	Wu et al., 2020
		miR-29a	TP53INP1 and NF-κB/NLRP3 axis	Liu et al., 2022a
**Spinal cord-related diseases**	Microglia	miR-151-3p miR-672-5p	p53/p21/CDK1 signaling pathway AIM2/ASC/Caspase-1 signaling pathway	Li et al., 2021 Zhou et al., 2022b
	Astrocytes	tRF-41	Wnt/ β-catenin signaling pathway	Cai et al., 2024
		APOE	LRP1	Jiang et al., 2024
	Schwann cells	Integrin-β	FAK/PI3K/Akt	Huang et al., 2022
		MFG-E8	SOCS3/STAT3 signaling pathway	Ren et al., 2023
		TLR2	NF-κB/PI3K signaling pathway	Pan et al., 2021
**Neurodegenerative diseases**	Microglia	miR-7670-3p miR-124-3p	ATF6 ROCK/PTEN/AKT/mTOR signaling pathway	Chen et al., 2023a Huang et al., 2021
**Peripheral nerve regeneration**	Schwann cells	SOX2 miR-21	FN1 SPRY2	Chen et al., 2022 Liu et al., 2022b

This table summarizes the roles of nerve cell-derived exosomes, including those from microglia, astrocytes, and Schwann cells, in various neurological disorders. The cargo of exosomes, such as microRNAs and proteins, regulates key pathways, thereby modulating neuroinflammation, apoptosis, axonal regeneration, and neuroprotection. These findings underscore the therapeutic potential of nerve cell-derived exosomes as novel cell-free strategies for intervening in neurological diseases. AIM2: Absent in melanoma 2; AKT: protein kinase B; ASC: apoptosis-associated speck-like protein containing a CARD; ATF4: activating transcription factor 4; ATF6: activating transcription factor 6; FAK: focal adhesion kinase; FN1: fibronectin 1; mTOR: mechanistic target of rapamycin; NF-κB: nuclear factor kappa-light-chain-enhancer of activated B cells; PI3K: phosphatidylinositol 3-Kinase; PTEN: phosphatase and tensin homolog; RGMa: repulsive guidance molecule A; ROCK: rho-associated coiled-coil containing protein kinase; Socsl: suppressor of cytokine signaling 1; SOCS3: suppressor of cytokine signaling 3; SOX2: SRY-box transcription factor 2; SPRY2: sprouty RTK signaling antagonist 2; STAT3: signal transducer and activator of transcription 3; TLR7: toll-like receptor 7; TP53INP1: tumor protein p53 inducible nuclear protein 1.

### Vascular system–derived exosomes

**Figure 5:**
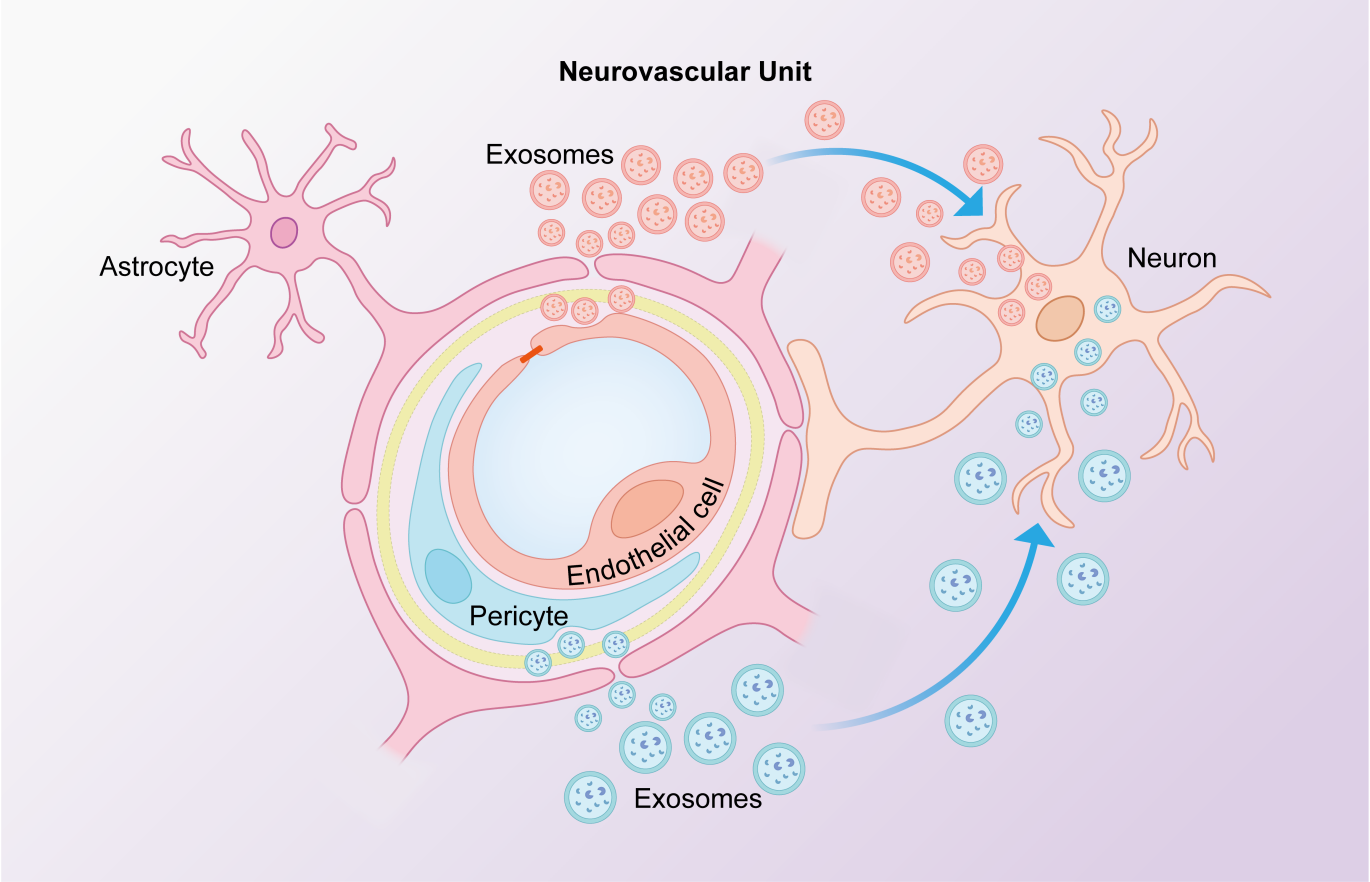
The neurovascular unit is a functional network consisting of vascular endothelial cells, pericytes, astrocytes, neurons, microglia, and the basement membrane. Within this network, vascular endothelial cells and pericytes secrete exosomes that play a role in neural regeneration and repair.

The neurovascular unit is a functional collaborative network in the CNS composed of multiple cells and structures, including neurons, astrocytes, microglia, endothelial cells, pericytes, and the basement membrane (Schaeffer and Iadecola, 2021). This unit maintains the normal metabolism and functional stability of the nervous system by preserving the integrity of the BBB and regulating neurovascular coupling and the inflammatory response. The integrity of the neurovascular unit is particularly important for nerve injury repair, as it provides essential support for the regeneration of damaged tissues by meeting the metabolic needs of neurons, regulating the inflammatory microenvironment, and promoting angiogenesis (Iadecola, 2017). During nerve regeneration, the neurovascular unit regulates blood supply through the cooperation of various cells and balances the expression of pro-inflammatory and anti-inflammatory factors to prevent excessive inflammation and further injury. The angiogenic function of the neurovascular unit supports nerve repair by facilitating the proliferation and migration of endothelial cells (Zhao et al., 2020b). However, dysfunction of the neurovascular unit, such as blood–brain barrier disruption or neurovascular signaling disorders, can significantly limit the effectiveness of nerve repair. In recent years, the potential role of exosomes as a key tool for intercellular signaling in regulating neurovascular unit function and promoting nerve regeneration has gradually become a research hotspot (**Figure 5**).

#### Endothelial cells

Endothelial cells, as a crucial component of the cardiovascular system, play a vital role in maintaining vascular homeostasis and contribute significantly to the function and repair of the nervous system (Trimm and Red-Horse, 2023). Endothelial cells improve tissue oxygen supply and nutrition by regulating angiogenesis, providing an optimized microenvironment for neural repair. Furthermore, their core position in the neurovascular unit indirectly supports neural regeneration by maintaining the integrity of the BBB and regulating the interaction between neurons and glial cells (Cong and Kong, 2020). In recent years, exosomes, a communication method mediated by EVs, have further demonstrated the function of endothelial cells in neural repair. Endothelial cell–derived exosomes are key messengers that endothelial cells use to orchestrate nerve regeneration and repair. Research has shown that these exosomes act as precise couriers, delivering miR-199-5p to Schwann cells, thereby enhancing their repair potential. This enhancement is reflected in increased proliferation, accelerated migration, and improved resistance to apoptosis. The driving force behind this repair mechanism is the PI3K/Akt/PTEN signaling pathway, which is activated by the miR-199-5p carried by exosomes. The activation of this pathway acts like pressing the accelerator, effectively promoting the repair process of peripheral nerve injury and quickly aiding in the restoration of function (Huang et al., 2023). In the CNS, exosomes derived from human umbilical vein endothelial cells pretreated with hypoxia also demonstrate significant efficacy. These exosomes are loaded with vascular endothelial growth factor and other angiogenic proteins, aimed at forming new capillary networks in the damaged area and optimizing microcirculation. This silent work provides essential logistical support for the regeneration of axons and the restoration of nerve function (Li et al., 2022). In summary, endothelial cell–derived exosomes are much more than mere construction tools for creating vascular networks; they are also an indispensable and versatile support force in the realm of nerve repair.

Further studies have found that endothelial cell–derived exosomes target and inhibit the expression of the angiogenesis inhibitor thrombospondin-1 by delivering miR-21-5p. This action has a dual effect: it enhances the self-repair ability of vascular endothelial cells and promotes the recovery of neurovascular unit function. This repair process requires the coordinated participation of signaling pathways, such as JAK/STAT and MAPK. Their joint regulation significantly reduces the inflammatory response in the nerve injury area and optimizes the supporting capacity of the local microenvironment (Gao et al., 2020a). In animal model experiments of cerebral ischemia–reperfusion injury, the therapeutic effect of endothelial cell–derived exosomes was independently verified; the treatment group exhibited a higher level of neuroplasticity, including reduced neuronal apoptosis, enhanced axonal regeneration, and accelerated neural network reconstruction (Hu et al., 2019). These findings provide a solid theoretical foundation for endothelial cell–derived exosome-based neural repair therapy, highlighting potential translational pathways.

Exosomes in the nervous system not only mediate intercellular communication mechanisms but also play a key regulatory role in maintaining myelin homeostasis, regulating neurogenesis, and ensuring the integrity of cognitive function (Fallahi et al., 2024). One study demonstrated that endothelial cell–derived exosomes can effectively enhance nerve regeneration and improve functional recovery by specifically transporting miRNA molecules, such as miR-126. miR-126 has been reported to be significantly enriched in endothelial cells and their exosomes, and it significantly improved the neurological prognosis of diabetic and stroke model mice by synergistically promoting axon extension and neovascularization (Venkat et al., 2019). In the field of cerebrovascular diseases, endothelial cell–derived exosomes also show significant neuroprotective effects. Experiments have confirmed that these exosomes can effectively regulate the information interaction network between endothelial cells and smooth muscle cells by delivering miR-204-5p molecules, thereby producing a protective regulatory effect in the pathological process of atherosclerosis (Tian et al., 2024). Additionally, one study revealed that exosomes can significantly alleviate the pathological damage of brain microvascular endothelial cells by bidirectionally regulating inflammatory cascades and oxidative stress levels (Yu et al., 2021). In summary, endothelial cell–derived exosomes achieve dual effects of neural regeneration and tissue protection in brain-related diseases through multidimensional regulatory mechanisms, including signaling pathway network regulation, promotion of angiogenesis, and remodeling of the inflammatory microenvironment.

Endothelial cell–derived exosomes are rich in miRNAs, such as miR-214, which play a key regulatory role in neuroprotection and angiogenesis. One study has shown that miR-214 inhibits cell aging and promotes angiogenesis through a dual mechanism, significantly enhancing the efficiency of signal transduction between endothelial cells (van Balkom et al., 2013). Notably, endothelial cell–derived exosomes can also directly regulate the survival status and functional activity of neurons by transporting specific proteins and lipid components. One study confirmed that endothelial cell–derived exosomes exhibit protective effects in models of myocardial ischemia–reperfusion injury by delivering sphingomyelin intermediate metabolites, such as sphingomyelin phosphorylcholine, suggesting that this mechanism may have potential applicability in nervous system protection (Yu et al., 2024). Further studies have revealed that these exosomes can also indirectly promote neuroregeneration by regulating the neuroinflammatory microenvironment and oxidative stress levels. Experimental data have shown that specific miRNAs effectively reduce the apoptosis rate and pathological damage in neurons by targeting and inhibiting the expression of inflammation-related factors (Xiao et al., 2017). In intervention strategies for neurodegenerative diseases, the functional regulation of neurovascular units by exosomes holds significant research value. It provides microenvironmental support for neural tissue regeneration and functional remodeling by maintaining a unique dynamic balance of neurovascular units (Forró et al., 2021). Typical examples show that EGR2 carried by exosomes upregulates SIRT6 to regulate the Notch signaling pathway, inhibiting programmed cell death while inducing angiogenesis, ultimately improving the level of neurological function recovery following ischemic stroke (Xiao et al., 2023). Comprehensive existing evidence demonstrates that endothelial cell–derived exosomes employ multidimensional molecular mechanisms, including miRNA signaling regulation, functional molecule delivery, neuroinflammation modulation, and oxidative stress microenvironment remodeling. These mechanisms collectively play a systemic protective role in the regeneration and repair processes associated with neurodegenerative diseases.

#### Pericytes

Pericytes are indispensable guardians of the microvascular wall. They are tightly attached to the outer surface of endothelial cells and, together with the basement membrane, form the core architecture of the neurovascular unit (Sweeney et al., 2016). The core responsibilities of these cells are concentrated on maintaining the stability of vascular structure, regulating the permeability of the BBB, and shaping the local microenvironment by releasing a variety of signaling molecules. Pericytes have made an indelible contribution to maintaining the homeostasis of the nervous system; they participate in the management of neurovascular coupling, ensure the supply of neural metabolism, and assist in the angiogenesis process through coordinated regulation with neurons, glial cells, and endothelial cells (Picoli et al., 2019). In particular, when neural tissue is damaged, pericytes can dedifferentiate and enter a highly active state, secreting cytokines and releasing exosomes to actively lay a more favorable foundation for the repair process of the damaged area (Cheng et al., 2018).

Pericyte-derived exosomes play a versatile role in the field of neural regeneration and repair. Their mechanism of action is closely linked to the precise activation and targeted regulation of multiple signaling pathways. One study has shown that pericyte-derived exosomes deliver miR-210-5p to vascular endothelial cells, significantly improving their functional status. This molecular key (miR-210-5p) successfully activates the JAK1/STAT3 signaling pathway, which, on one hand, enhances the activity of mitochondria and, on the other hand, inhibits harmful lipid peroxidation reactions, jointly protecting the integrity of the blood-brain barrier after spinal cord injury. Thanks to the regulation of these pathways, pericyte-derived exosomes effectively drive the proliferation and migration of vascular endothelial cells, optimize microcirculation in the injured area, and deliver the necessary blood supply and nutritional support for neural repair (Gao et al., 2023). Furthermore, pericyte-derived exosomes can actively upregulate the expression of key angiogenic factors (such as vascular endothelial growth factor and Ang1), accelerate the construction of new vascular networks, and create a more fertile environment for the reconstruction and functional recovery of nerve axons (Yin et al., 2022).

In the repair of peripheral nerve injury, pericyte-derived exosomes exhibit significant neuroprotective effects. These exosomes provide a cellular basis for the regeneration of injured nerve fibers by enhancing the repair phenotype of Schwann cells, including their proliferation, migration, and axon guidance abilities. This effect is primarily achieved by activating the PI3K/Akt signaling pathway, accompanied by the upregulation of neurotrophic factors such as brain-derived neurotrophic factor and Nerve growth factor. Additionally, pericyte-derived exosomes can inhibit the JNK/c-Jun signaling pathway, significantly reducing the level of cell apoptosis (Ock et al., 2023). Collectively, these molecular actions have been shown to accelerate functional recovery after sciatic nerve injury and significantly improve the motor and sensory abilities of mice.

In addition to acting directly on nerve cells, pericyte-derived exosomes promote nerve regeneration indirectly by regulating the immune microenvironment. Studies have shown that these exosomes can induce macrophage polarization to an anti-inflammatory phenotype, reduce the release of pro-inflammatory factors, and thereby alleviate local inflammatory responses. This immunomodulatory effect further optimizes the microenvironment for nerve repair and promotes the regeneration of damaged tissues. Particularly in the study of spinal cord injury, pericyte-derived exosomes demonstrated strong neuroprotective and regeneration-promoting effects by acting on vascular endothelial cells, Schwann cells, and immune cells (Yuan et al., 2019). In summary, pericyte-derived exosomes participate in the regulation of nerve regeneration and repair through multiple mechanisms. Their scope of action encompasses various aspects, including angiogenesis, neuroprotection, immune regulation, and inflammation control (**Additional Table 4**).

**Additional Table 4:** Role of vascular cell-derived exosomes in neurological diseases

Neurological disorders	Source of exosomes	Cargo	Molecular target	Reference
**Brain-related diseases**	Endothelial cells	miR-126	PTPN9	Zhou et al., 2020
		miR-126-3p	Synapsin-I	Gao et al., 2020a
		miR-21-5p	THBS1	Hu et al., 2019
		miR-126	SPRED1	Venkat et al., 2019
		miR-589-5p	TET2	Yu et al., 2021
		SPC	NR4A2/OPTN signaling pathway	Yu et al., 2024
**Spinal cord-related diseases**	Endothelial cells	miR-199-5p	PI3K/AKT/PTEN signaling pathway	Huang et al., 2023
	Pericyte	miR-210	JAK1/STAT3 signaling pathway	Gao et al., 2023
		BDNF	PI3K/AKT/PTEN signaling pathway	Yin et al., 2022
		Hebp1	claudin	Ock et al., 2023

This table summarizes the roles of vascular cell-derived exosomes, including those secreted by endothelial cells and pericytes, in various neurological disorders. The cargos of these exosomes act on specific targets, thereby modulating synaptic function, neuroinflammation, angiogenesis, and blood-brain barrier integrity. These findings highlight the crucial role of vascular cell-derived exosomes in neural repair and their potential as therapeutic tools for neurological diseases. AKT: Protein kinase B; BDNF: brain-derived neurotrophic factor; Hebp1: heme binding protein 1; JAK1: Janus kinase 1; NR4A2: nuclear receptor subfamily 4 group A member 2; OPTN: optineurin; PI3K: phosphatidylinositol 3-kinase; PTEN: phosphatase and tensin homolog; PTPN9: protein tyrosine phosphatase non-receptor type 9; SPC: surfactant protein C; SPRED1: sprouty related EVH1 domain containing 1; STAT3: signal transducer and activator of transcription 3; TET2: tet methylcytosine dioxygenase 2; THBS1: thrombospondin 1.

A growing body of research demonstrates the functional heterogeneity of exosomes derived from different tissues and cells. This diversity primarily arises from the cargo they carry, including microRNAs and proteins. These bioactive components act on specific molecular targets and signaling pathways, playing diverse roles in regulating neuronal survival, neuroinflammation, axonal regeneration, and myelin regeneration in neurological diseases. To comprehensively review current research findings, **Additional Table 5** summarizes representative exosome cargo molecules from various tissues, their targets, and the potential signaling mechanisms involved in disease progression and repair.

**Additional Table 5:** Exosomes derived from different tissues deliver specific cargos that act through distinct molecular mechanisms in neurological diseases

Category	Cargo	Mechanism in the nervous system	Reference
miRNAs	miR-21	It inhibits SPRY2 expression, which relieves the inhibition of axonal growth and promotes the expression of GAP43 and DNMT3A, thereby enhancing neuronal axonal growth and synaptic plasticity.	Liu et al., 2022b
	miR-22-3p	It activates the mTORCl signaling pathway, promoting axon regeneration and retinal ganglion cell (RGC) survival by regulating protein synthesis and genes related to cell growth.	Sang et al., 2023
	miR-22-3p	It inhibits PTEN expression, which relieves the inhibition of the AKT/mTOR signaling pathway, thereby enhancing the proliferation and migration of Schwann cells and promoting axon growth in dorsal root ganglion neurons.	Yang et al., 2022
	miR-23a-3p	It inhibits Tbr1, and its downstream non-canonical Wnt signaling relieves the suppression of myelin-related protein expression and activates the PI3K/Akt pathway, promoting cell differentiation and survival.	Qin et al., 2024
	miR-23b	It modulates the PTEN/Nrf2 antioxidant pathway and the PTEN/NLRP3 inflammasome pathway to alleviate oxidative stress and neuroinflammation after intracerebral hemorrhage.	Hu et al., 2023a
	miR-23b-3p	It inhibits NEK7 expression and blocks its interaction with the NLRP3 inflammasome, thereby attenuating NLRP3 inflammasome activation and reducing Caspase-1-mediated pyroptosis and the release of pro-inflammatory cytokines.	Wang et al., 2023
	miR-26a	It downregulates PTEN expression and activates the AKT/mTOR signaling pathway, thereby promoting neuronal and axonal regeneration and alleviating glial scar formation.	Chen et al., 2021
	miR-26a-5p	It inhibits the expression of CDK6, reduces the apoptosis level of microglia and alleviates the scope of brain ischemic damage.	Cheng et al., 2021
	miR-29a	It targeted inhibition of TP53INP1 and regulation of the NF-κB/NLRP3 signaling pathway effectively alleviates oxidative stress damage and inhibits inflammatory response.	Liu et al., 2022a
	miR-29c-3p	It downregulates BACE1 expression, thereby reducing the generation and deposition of Aβ, while activating the Wnt/β-catenin signaling pathway, enhancing neuronal vitality and inhibiting apoptosis.	Sha et al., 2021
	miR-34c	It inhibits TLR7 expression and downregulates the NF-κB/MAPK inflammatory pathway, thereby alleviating neuronal damage caused by ischemia-reperfusion injury, promoting neuronal proliferation and inhibiting apoptosis.	Wu et al., 2020
	miR-124	It inhibits cell apoptosis and promotes neuronal survival by downregulating its target gene USP14.	Song et al., 2019
	miR-124-3p	It inhibits MYH9 expression, activates the PI3K/Akt pathway, and inhibits the NF-κB signaling cascade, effectively suppressing the activation of M1 microglia and A1 astrocytes.	Jiang et al., 2020
	miR-126	It significantly promotes angiogenesis, myelin and axonal remodeling, and induces macrophage polarization to the M2 type, ultimately improving neurological function and cognitive recovery.	Venkat et al., 2019
	miR-126-3p	It can be taken up by neurons to inhibit apoptosis and promote the growth of axons and dendrites, while also upregulating synapsin-I protein and enhancing synaptic plasticity.	Gao et al., 2020a
	miR-128-3p	It downregulates ACVR1 expression, blocks BMP signaling activation, promotes MBP expression and myelin repair, thereby reducing infarct volume and improving neurological recovery.	Hou et al., 2023
	miR-145	It directly targets and downregulates FOXO1, thereby reducing apoptosis, cell cycle arrest, and oxidative stress in microglia under OGD/R treatment and promoting their polarization toward the anti-inflammatory M2 type.	Zhou et al., 2022a
	miR-146a	It significantly downregulates the expression of IRAKI and TRAF6, blocks NF-κB p65 activation, thereby reducing the release of inflammatory factors and alleviating oxidative stress.	Tang et al., 2024
	miR-146a-5p	It inhibits the TRAF6/IRAK1-mediated NF-κB signaling cascade, significantly reduces the activation of neurotoxic astrocytes and the release of inflammatory factors, alleviates neuronal damage and protects neural tissue.	Lai et al., 2022
	miR-148b-3p	It alleviates cerebral ischemia-reperfusion injury by inhibiting the DLL4/Notch1 signaling pathway, reducing microglial activation, proliferation, migration and secretion of inflammatory factors.	Yi et al., 2024
	miR-150-3p	It downregulates the expression of CASP2, thereby inhibiting pro-apoptotic factors such as Bax and Caspase-3 and up-regulating Bcl-2, significantly reducing neuronal apoptosis, promoting neuronal proliferation and oligodendrocyte differentiation.	Luo et al., 2022
	miR-151-3p	It downregulates p53 expression and inhibits the p53/p21/CDK1 pathway, thereby reducing the expression ofpro-apoptotic proteins such as Bax and cleaved-caspase-3 and up-regulating Bcl-2. Ultimately, this reduces neuronal apoptosis and promotes axonal regeneration.	Li et al., 2021
	miR-155-5p	It leads to downregulation of Socs1 expression, thereby relieving its inhibition of NF-κB, promoting the inflammatory cascade and inducing M0 to M1 phenotype polarization.	Chen et al., 2023b
	miR-199-5p	It activates the PI3K/AKT/PTEN pathway, thereby promoting the repair phenotype transformation of SCs, enhancing their proliferation and migration, and accelerating axon regeneration.	Huang et al., 2023
	miR-199a-5p	It downregulates GSK-3β expression, relieves its inhibition on β-catenin, promotes β-catenin nuclear translocation and upregulates Cyclin D1, thereby enhancing NSC proliferation.	Yang et al., 2023
	miR-210	It downregulates JAK1 expression and inhibits JAK1/STAT3 signaling activity, thereby improving mitochondrial function, suppressing lipid peroxidation and oxidative stress, and enhancing the integrity of the blood-spinal cord barrier.	Gao et al., 2023
	miR-214-3p	circRNA Nkd2 relieves the inhibition of miR-214-3p on MED19, promotes the proliferation of Schwann cells, and thus accelerates remyelination, axonal regeneration, and functional recovery after facial nerve injury.	Wang et al., 2024
	miR-223-3p	It inhibits microglial M1 polarization and promotes M2 transformation, and it exerts anti-inflammatory and neuroprotective effects by targeting and inhibiting CysLT2R.	Zhao et al., 2020a
	miR-367-3p	It inhibits microglial ferroptosis by inhibiting EZH2 and upregulating the SLC7A11/GPX4 pathway.	Fan et al., 2023
	miR-431-3p	It inhibits the expression of RGM-A, relieves its inhibition on axon growth, and significantly promotes axon extension, branching, and synapse formation.	Sun et al., 2024
	miR-501-5p	It inhibits MLCK expression, stabilizing tight junction proteins, reducing blood-spinal cord barrier permeability, and promoting axonal regeneration.	Xie et al., 2023
	miR-589-5p	Overexpression of circ_0003423 can absorb miR-589-5p, thereby relieving its inhibition on TET2 and increasing TET2 expression, thereby inhibiting cell apoptosis and improving cell viability.	Yu et al., 2021
	miR-672-5p	It directly targets and inhibits E2F1 expression, thereby downregulating its pro-apoptotic pathway, reducing neuronal apoptosis and improving cell viability.	Zhou et al., 2022b
	miR-5121	It inhibits RGMa, promoting neuronal axonal growth and synaptic plasticity, thereby improving functional recovery after TBI.	Zhao et al., 2021a
	miR-7670-3p	It inhibits ATF6 expression, alleviates endoplasmic reticulum stress and inflammatory response, reduces AP deposition, and protects dendritic spines and synaptic plasticity.	Chen et al., 2023a
Proteins	BDNF	It increases the expression level of MAP2, promotes the differentiation ofneural progenitor cells into neurons, and enhances the ability of neurogenesis.	Zhou et al., 2020
	BDNF	It acts on olfactory ensheathing cells, activates BDNF/TrkB signaling, thereby improving the migration ability of OECs and significantly promoting axon regeneration.	Zhang et al., 2020a
	BDNF	It activates PI3K/Aktto promote cell survival and inhibits JNK/c-Jun-mediated apoptosis, thereby promoting angiogenesis and nerve regeneration.	Yin et al., 2022
	BDNF and NGF	These proteins promote the proliferation and axonal growth ofdorsal root ganglion neurons and Schwann cells, and enhance the regeneration capacity of peripheral nerves.	Bucan et al., 2019
	Hebpl	It interacts with claudin-2/3 through Hebpl, regulates tight junction protein expression, reduces vascular permeability and oxidative stress levels, and thus promotes neurovascular regeneration.	Ock et al., 2023
	HGF, BDNF and TNFR1	These proteins regulate MDA and T-SOD levels, improve oxidative stress, and significantly alleviate cerebral ischemia-reperfusion injury in rats.	Liu et al., 2024a
	MG53	It regulates MDA, SOD, and ROS levels by activating the Nrf2 signaling pathway (upregulating Nrf2, NQO1, and SOD1, and downregulating Keap-1).	Ma et al., 2022
	MFG-E8	It regulates the SOCS3/STAT3 signaling pathway, promotes the transformation of macrophages/microglia from M1 to M2, and reduces neuronal apoptosis.	Ren et al., 2023
	GJA1-20k	It reduces cell apoptosis (downregulation of Bax, Caspase-3, cytoC, upregulation of Bcl-2; restores mitochondrial function and structural integrity, and improves neuronal dendritic morphology.	Chen et al., 2020
	APOE	It can reduce microglial activation and inflammatory factor release by binding to its receptor LRP1, inhibiting demyelination and brain damage.	Jiang et al., 2024
	TLR2	It inhibits CSPG deposition through the NF-kB/PI3K signaling pathway, thereby improving the axonal regeneration environment and promoting nerve function recovery.	Pan et al., 2021
	SOX2	It inhibits CSPG deposition through the NF-κB/PI3K signaling pathway, thereby improving the axonal regeneration environment and promoting nerve function recovery.	Chen et al., 2022

ACVR1: Activin A receptor type 1; AKT: protein kinase b; APOE: apolipoprotein E; ATF6: activating transcription factor 6; Aβ: amyloid-beta; BACE1: beta-secretase 1; Bax: bcl2-associated x protein; Bcl-2: b-cell lymphoma 2; BDNF: brain-derived neurotrophic factor; BMP: bone morphogenetic protein; CASP2: caspase 2; caspase-1: cysteinyl aspartate specific proteinase-1; caspase-3: cysteinyl aspartate specific proteinase-3; CDK1: cyclin-dependent kinase 1; CDK6: cyclin-dependent kinase 6; circRNA: circular RNA; CSPG: chondroitin sulfate proteoglycan; cytoC: cytochrome c; CysLT2R: cysteinyl leukotriene receptor 2; DLL4: delta like canonical notch ligand 4; DNMT3A: DNA methyltransferase 3 alpha; E2F1: e2f transcription factor 1; EZH2: enhancer ofzeste 2 polycomb repressive complex 2 subunit; FOXO1: forkhead boxo1; GAP43: growth associated protein 43; GJA1-20k: gap junction alpha-1 protein, 20kda isoform; GPX4: glutathione peroxidase 4; GSK-3β: glycogen synthase kinase-3 beta; Hebp1: heme binding protein 1; HGF: hepatocyte growth factor; IRAK1: interleukin-1 receptor associated kinase 1; JAK1: j anus kinase 1; JNK: c-jun n-terminal kinase; Keap-1: Kelch-like ech-associated protein 1; LRP1: low-density lipoprotein receptor-related protein 1; MAP2: microtubule-associated protein 2; MAPK: mitogen-activated protein kinase; MBP: myelin basic protein; MDA: malondialdehyde; MED19: mediator complex subunit 19; MFG-E8: milk fat globule-epidermal growth factor 8; MG53: mitsugumin 53; miRNA: microrna; MLCK: myosin light chain kinase; mTOR: mammalian target of rapamycin; mTORC1: mammalian target of rapamycin complex 1; MYH9: myosin heavy chain 9; NEK7: nima related kinase 7; NF-κB: nuclear factor kappa-light-chain-enhancer of activated b cells; NGF: nerve growth factor; NLRP3: nlr family pyrin domain containing 3; NQO1: nad(p)h quinone dehydrogenase 1; Nrf2: nuclear factor erythroid 2-related factor 2; NSC: neural stem cell; OECs: olfactory ensheathing cells; OGD/R: oxygen-glucose deprivation/reoxygenation; PI3K: phosphatidylinositol 3-kinase; PTEN: phosphatase and tensin homolog; RGC: retinal ganglion cell; RGMa: repulsive guidance molecule a; ROS: reactive oxygen species; SCs: Schwann cells; SLC7A11: solute carrier family 7 member 11; SOD: superoxide dismutase; SOD1: superoxide dismutase 1; Socs1: suppressor of cytokine signaling 1; SOX2: sry-box transcription factor 2; SPRY2: sprouty RTK signaling antagonist 2; STAT3: signal transducer and activator of transcription 3; T-SOD: total superoxide dismutase; TBI: traumatic brain injury; Tbr1: t-box brain transcription factor 1; TET2: tet methylcytosine dioxygenase 2; TLR2: toll-like receptor 2; TLR7: toll-like receptor 7; TNFR1: tumor necrosis factor receptor 1; TP53INP1: tumor protein p53 inducible nuclear protein 1; TRAF6: TNF receptor associated factor 6; TrkB: tropomyosin receptor kinase b; USP14: ubiquitin specific peptidase 14; Wnt: wingless/integrated.

## Current Status of Clinical Translation and Application

Clinical translational research on EVs is gradually becoming an important focus in the biomedical field. Recent studies have found that the unique intercellular communication mechanisms and bioactive molecule delivery functions of EVs provide a theoretical foundation for their application in disease diagnosis and treatment (Ozkocak et al., 2022). Currently, the academic community has reached a consensus: EVs possess dual value as both new diagnostic markers and therapeutic delivery carriers (Rädler et al., 2023; Yao et al., 2023). The global clinical trial registration database shows that the number of EV-related research programs has increased exponentially, reflecting the growing international attention to their clinical translational value (**Box 1**). In the study of neurological diseases, the potential application of EVs in treating neurodegenerative diseases and acute nerve injuries has garnered considerable attention. One study has shown that MSC-derived EVs demonstrate significant neuroprotective effects in models of ischemic stroke, and their safety and efficacy have progressed to clinical verification (Lotfy et al., 2023). Notably, the EV-based therapeutic molecule delivery system has made breakthroughs in Alzheimer’s disease intervention research, providing a new strategy for delaying disease progression. The field of tumor treatment is exploring the dual application of EVs, which can function as both an efficient drug delivery system and a direct therapeutic agent. Recent clinical trial data indicate that tumor-derived EVs exhibit good biocompatibility in the treatment of non-small cell lung cancer, and research into their anti-tumor activity mechanisms has yielded promising results. It is particularly noteworthy that innovative clinical research on EVs in cardiovascular diseases and autoimmune diseases is accelerating, and the academic community generally recognizes their multi-field application prospects.

Box 1: Progress in clinical translation of the researches in artificial hibernation technology

**(1) Clinical trials of extracellular vesicles and their safety evaluation**

a. In recent years, a total of 12 extracellular vesicles (EVs)-related neurological studies (9 interventional studies and 3 observational studies) have been included on the ClinicalTrials.gov website, primarily addressing neurodegenerative diseases, acute ischemic stroke, refractory focal epilepsy, neuropathic pain, and traumatic brain injury. The interventions primarily consist of exosomes derived from mesenchymal stem cells (MSC) or induced pluripotent stem cells (iPSC), which are delivered either intranasally or through injection. The majority of these studies are in the early phases and focus on assessing safety and initial efficacy, indicating a promising potential for translation into clinical practice.

b. Recently registered or actively enrolling studies demonstrate an expansion in indications and dosing routes. For example, NCT05886205 is an early Phase 1 trial investigating the use of iPSC exosome nasal drops for treating refractory focal epilepsy. NCT06138210 is a Phase 1 trial examining the comparison between iPSC exosome injections and a placebo in patients with acute ischemic stroke. NCT06607900 is another Phase 1 trial focusing on neurodegenerative diseases, utilizing human umbilical cord mesenchymal stem cell-derived small extracellular vesicles (hUC-MSC-sEV-001) delivered via nasal drops. Lastly, NCT05152368 is a Phase 1 trial exploring the use of AlloEx for peripheral neuropathy and trigeminal neuralgia. These studies highlight the importance of blood–brain barrier-permeable intranasal delivery, parenteral administration, and a continuous emphasis on safety and feasibility endpoints.

**(2) Approval from the U.S. Food and Drug Administration**

The U.S. Food and Drug Administration (FDA) has increasingly focused on the application of EVs in the new drug approval process. The FDA is still in the exploratory stage of regulating EV-related products, with no EV-based therapeutic products currently approved. However, many studies have progressed to the clinical trial new drug application (IND) stage.

**(3) The financial support for the National Institutes of Health**

a. The National Institutes of Health (NIH) has funded 10 projects focusing on extracellular vesicles for the treatment of traumatic brain injury, stroke, and neuroinflammation, as well as engineered extracellular vesicles for central nervous system (CNS) barrier delivery, CNS-derived exosome biomarkers for neonatal hypoxic-ischemic encephalopathy, and the underlying mechanisms. These research results are expected to provide important references for future clinical practice, indicating that the federal government’s support is both sustained and diverse.

b. These awards collectively emphasize therapeutic EV engineering and CNS translation, aligning with the clinical pipeline’s focus on acute neural injury and CNS indications, and providing programmatic momentum for future trials.

The U.S. Food and Drug Administration has increasingly focused on the application of EVs in the new drug approval process. The U.S. Food and Drug Administration is still in the exploratory stage of regulating EV-related products, with no EV-based therapeutic products currently approved; however, many studies have entered the Investigational New Drug application stage (Verdin, 2024). For example, EXO-CD24, a new therapy that combines the CD24 protein with exosomes, is being tested in clinical trials for COVID-19 in Israel and has shown positive efficacy and safety, laying the groundwork for further development of EVs in clinical applications (Miller and Miller Needleman, 2023). The role of the National Institutes of Health in funding EV research cannot be overlooked. In recent years, the National Institutes of Health has funded several studies on EVs in the treatment of neurological diseases, covering multiple levels, from basic mechanism research to preclinical and clinical studies (**Box 1**). The implementation of these funding projects will help promote the application of EVs in the field of neural regeneration and repair. In terms of patents, the basic research and technological development of EVs continue to deepen. In recent years, the number of global EV-related patent applications has grown exponentially, with core technologies involving key areas such as efficient separation and purification processes, targeted drug delivery system development, and engineering transformation technology (Sadeghi et al., 2023). Notably, EVs have shown unique advantages in the targeted therapy of malignant tumors and in the immune regulation of infectious diseases (Kimiz-Gebologlu and Oncel, 2022). The increase in these patents reflects the international academic community’s growing attention and expectations for the clinical transformation value of EVs.

In summary, EVs have demonstrated remarkable potential for clinical applications. Although there are still technical bottlenecks at this stage, with breakthrough progress in molecular characterization technology and the gradual improvement of the regulatory system, EVs will undoubtedly play a more significant role in the field of precision medicine.

## Biosafety Evaluation

As a new type of therapeutic carrier, EVs have become increasingly prominent in the treatment of neurological diseases. However, the biosafety issues associated with EVs, described as a “double-edged sword,” need to be systematically analyzed. The first dimension to consider involves the immunomodulatory properties of EVs, particularly the potential sensitization of their surface antigens (Shakhnovich et al., 2020). Although EVs benefit from biocompatibility due to the characteristics of the donor cell membrane, in heterologous delivery scenarios, the foreign antigen epitopes presented on their membrane surface may trigger abnormal activation of the host immune system (Shi et al., 2021). This immune cascade reaction may not only induce a local inflammatory microenvironment but also affect the efficiency of the therapeutic response through immune checkpoint interference.

Another key risk dimension is the potential neurotoxic effect of EVs. EVs secreted under pathological conditions may be enriched with neurotoxic factors, such as misfolded proteins or non-coding RNAs, which can lead to changes in synaptic plasticity or even neurodegeneration through abnormal signal transduction (Xu et al., 2016). It is important to note that EVs may play a dual regulatory role in the pathological process of tumor microenvironment reconstruction, and the bioactive molecules they carry may serve as molecular carriers for the transmission of malignant phenotypes. Existing evidence demonstrates that the EV-mediated horizontal transfer of oncogenes and proteins can promote the epithelial-mesenchymal transition process in tumor cells through epigenetic reprogramming. Its potential genotoxicity risk should not be overlooked. The genetic information carried in EVs may interfere with the stability of the host genome through mechanisms such as retrotransposition, leading to genotoxic events like chromosomal structural variations. Furthermore, the unique nanoscale penetration characteristics of EVs provide an advantage for crossing the central nervous system (CNS), but this biological characteristic may also result in off-target risks due to nonspecific distribution, potentially causing functional disorders in remote organs (Debbi et al., 2022).

In the future, improving the safety of EVs in the treatment of neurological diseases will require a systematic research strategy that emphasizes multidisciplinary collaboration. The first task is to analyze the biological characteristics of EVs in detail, including their components, mechanisms of action, and dynamic interactions with the host immune system; this analysis will serve as the theoretical basis for controlling their potential adverse reactions. Establishing a standardized process for EV preparation and purification to ensure batch-to-batch consistency and high purity is crucial for reducing immunogenicity and toxicity risks. By integrating organoid culture systems and organ-on-a-chip technology to create biomimetic models, the efficacy and safety characteristics of EVs can be evaluated more accurately (Zhang et al., 2020b). Ultimately, it is essential to enhance the translational research system from preclinical to clinical stages and establish an evidence-based medicine framework by accumulating multidimensional safety data.

## Limitations

This review systematically examines the molecular mechanisms and functional characteristics of exosome-mediated neural regeneration, focusing on its clinical application prospects in the field of neural repair. Although exosomes can cross the blood–brain barrier, their *in vivo* delivery efficiency and tissue targeting accuracy remain key bottlenecks restricting clinical translation. It is worth noting that most current mechanism studies are still limited to *in vitro* experimental model systems, lacking *in vivo* functional verification and physiological barrier penetration studies. At the same time, circulating brain-derived EVs have shown diagnostic potential as liquid biopsies in revealing the pathology of CNS diseases. However, the molecular cargo they carry may not necessarily reflect the molecular composition of the parent cells, and caution should be exercised in the biological interpretation of related indicators. A key challenge in studying EVs crossing the blood–brain barrier, whether *in vivo* or *in vitro*, lies in visualization. The particle size of EVs often approaches the diffraction limit of light microscopy (Verweij et al., 2021). Therefore, the resulting resolution constraints must be considered and accounted for in experimental design and data interpretation. Currently, the pharmacokinetic characteristics of exosomes (including metabolic pathways, tissue distribution patterns, and clearance mechanisms) have not been systematically elucidated, and their biological effects may be influenced by donor heterogeneity, showing significant individual differences (Barile and Vassalli, 2017). In light of this, it is urgent to develop intelligent delivery systems to achieve precise regulation of exosome spatial positioning and enhance therapeutic efficacy through molecular engineering strategies, such as targeted antibody functional modification and genome editing regulation (Xi et al., 2021).

Existing clinical evidence exhibits significant cross-study heterogeneity due to differences in cell source, preparation/isolation, and route of administration, making it difficult to assert an overall superiority in efficacy for any specific exosome subtype or cell source. Regarding safety, a recent meta-analysis of 21 interventional clinical studies (*n* = 335) demonstrated an overall incidence of serious adverse events of only 0.7%, with no significant differences in serious adverse events between autologous and allogeneic, or engineered and non-engineered products (Van Delen et al., 2024). However, these results cannot be used to determine the superiority of efficacy, as significant methodological variability precludes cross-sectional comparisons. Current clinical studies also rarely stratify systematically by specific payload type. The aforementioned meta-analysis, which only sub-grouped by “engineered *vs.* non-engineered,” showed no increase in serious adverse events with engineered products; however, a higher rate of adverse event reporting remains insufficient to infer clinical differences between different payloads. To enhance comparability, the standardized recommendations of the Minimal Information for Studies of EVs 2023 regarding separation, characterization, and dose reporting should be followed. Additionally, stratified randomization and potency assessment by loading category should be predefined in clinical trials. Most existing studies are early-stage trials and often use repeated dosing, lacking direct comparisons with single dosing, and the durability of efficacy remains uncertain. Future studies should systematically report efficacy-time curves and minimum effective doses to clarify the duration of effectiveness.

In summary, although exosomes present revolutionary therapeutic prospects in the field of neural regeneration, they must overcome systemic obstacles such as a lack of technical standardization, limited applicability of cross-species models, poor efficacy of targeted delivery systems, and obstructed clinical transformation paths (Gutierrez-Millan et al., 2021; Xu et al., 2023a). Subsequent research needs to focus on building a three-level technical system: optimizing the preparation process and standardized purification process through molecular engineering strategies, using multi-omics technology to create multidimensional mechanism maps, and developing intelligent responsive delivery systems. The synergy of these three aspects will significantly enhance the clinical transformation value of exosomes. It is particularly important to emphasize that establishing a multicenter clinical trial network based on evidence-based medicine, combined with a whole-life-cycle safety monitoring platform, will be essential for the clinical transformation of exosome therapy (EL Andaloussi et al., 2013; Batrakova and Kim, 2015; Tenchov et al., 2022). Only by systematically overcoming these scientific challenges can exosomes achieve a breakthrough in the treatment paradigm shift for neural regeneration and ultimately develop a precise and personalized functional reconstruction solution for patients with neural injuries.

## Conclusion

Neuroregeneration is a major challenge in the field of neuroscience, particularly concerning nerve injury, degenerative diseases, and the reconstruction of the neural microenvironment. Although modern medicine has made significant progress in nerve repair, many challenges remain. The difficulty of repairing nerves after injury and the lack of effective treatments for neurodegenerative diseases render the process of nerve regeneration fraught with uncertainty. To address these challenges, current researchers are actively exploring innovative therapies that can effectively promote nerve regeneration. EVs have shown great potential in this area in recent years, playing an important role in the treatment of CNS diseases through various mechanisms, such as regulating neuroinflammation and promoting nerve repair.

This review systematically examines the role of EVs in neuroregeneration and highlights their potential applications in the field of neuroregenerative science. As natural intercellular signaling vehicles, EVs can carry a variety of bioactive molecules (such as proteins, RNAs, and microRNAs). These molecules contribute to the repair of neural damage by influencing interactions between neurons and glial cells. Furthermore, the regulatory effects of EVs within the neural microenvironment can provide important support for neural regeneration. These properties offer significant clinical advantages for EVs in the treatment of neural injuries and neurodegenerative diseases, particularly those requiring long-term treatment, such as low immunogenicity and good biocompatibility.

However, most existing research focuses on EVs from a specific source, overlooking the diversity and complexity of their origins. EVs from different sources exhibit significant differences in composition and function, which may influence the effectiveness of neural repair. Current research has rarely considered how different types of EVs work synergistically during neural regeneration, which limits our comprehensive understanding of the role of EVs in neural repair. Therefore, it is crucial to propose a comprehensive framework to integrate the mechanisms of action of EVs from different sources in neural regeneration (**Additional Table 5**). Within this framework, we can focus on how EVs regulate neural regeneration through precise molecular mechanisms. EVs transmit signals through direct interactions with target cells and act remotely to alter the properties of the neural microenvironment, creating more favorable conditions for neural regeneration. Through this multi-layered regulatory mechanism, the role of EVs in neural regeneration may achieve a wider range of therapeutic benefits.

This review systematically explores the potential of EVs in neurorepair and provides new perspectives for their clinical translation. Traditional neuroregenerative therapeutic strategies have largely focused on drug interventions or cell therapies, but these approaches are often associated with significant side effects or limited efficacy. In contrast, EVs, as natural delivery vehicles, exhibit significant potential in neuroregenerative therapies due to their low immunogenicity and excellent biocompatibility. In particular, their ability to cross the CNS and accommodate long-term treatment needs offers new therapeutic avenues for neurological diseases.

However, despite their promising applications, clinical translation of EVs faces numerous challenges. The current separation and purification technologies for EVs still need improvement. The existing extraction methods are diverse and must strictly adhere to international guidelines. However, different methods often show considerable differences in separation efficiency, purity, and yield. Therefore, optimizing the preparation process is critical to enhancing the therapeutic effect. Additionally, existing preclinical studies are mostly based on mouse and rat models, which possess strong nerve regeneration capabilities and differ from human physiology. This discrepancy may limit the reliability of their conclusions for clinical translation. Thus, it is essential to systematically evaluate the mechanisms of action and stability of EVs in a wider variety of nerve injury models, especially under chronic pathological conditions.

Future research should involve an in-depth analysis of the multidimensional regulatory networks of EVs, integrating multi-omics approaches such as single-cell RNA sequencing and proteomics to explore these issues. Meanwhile, the delivery efficiency and targeting of EVs remain major challenges in their clinical application. Although they demonstrate a natural ability to cross the CNS, their *in vivo* delivery efficiency and targeting accuracy are still limited. Therefore, efforts should be made to develop intelligent delivery systems that combine real-time tracking with precise controllability to enhance their therapeutic efficacy and facilitate clinical translation.

As an innovative therapeutic strategy for neuroregeneration, EVs demonstrate broad application prospects. With continued technological advancement and in-depth interdisciplinary collaboration, the potential for clinical translation of these vectors in the field of neuroregeneration is highly anticipated. Future research should focus on achieving breakthroughs in key areas such as standardized preparation, preclinical validation, and safety evaluation to promote the practical application of EVs in the treatment of central nervous system diseases and ultimately achieve the goal of precision therapy.

## Additional files:

***Additional Table 1:*** *EV nomenclature.*

***Additional Table 2:*** *Role of exosomes derived from mesenchymal stem cells in neurological diseases.*

***Additional Table 3:*** *Role of nerve cell–derived exosomes in neurological diseases.*

***Additional Table 4:*** *Role of vascular cell–derived exosomes in neurological diseases.*

***Additional Table 5:*** *Exosomes derived from different tissues deliver specific cargos that act through distinct molecular mechanisms in neurological diseases.*

## Data Availability

*All relevant data are within the paper and its Additional files.*
